# Supplementary figures and images for: Bioinformatics and modelling studies of FhuD, the periplasmic siderophore binding protein from the plant pathogen Erwinia amylovora
Source: PLoS One. 2025 Jul 23;20(7):e0326667. doi: 10.1371/journal.pone.0326667 (PMC12286361; doi:10.1371/journal.pone.0326667)

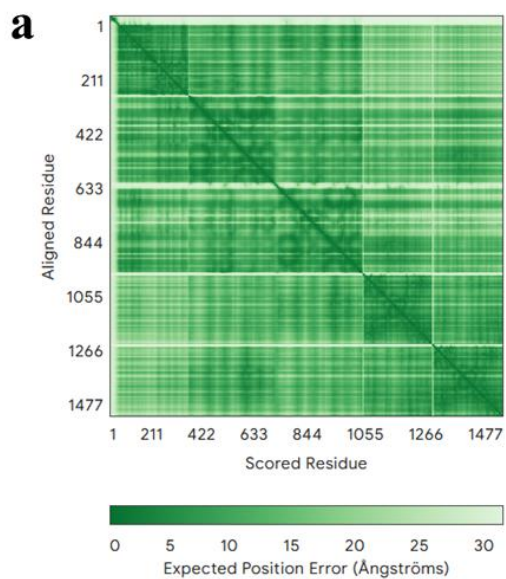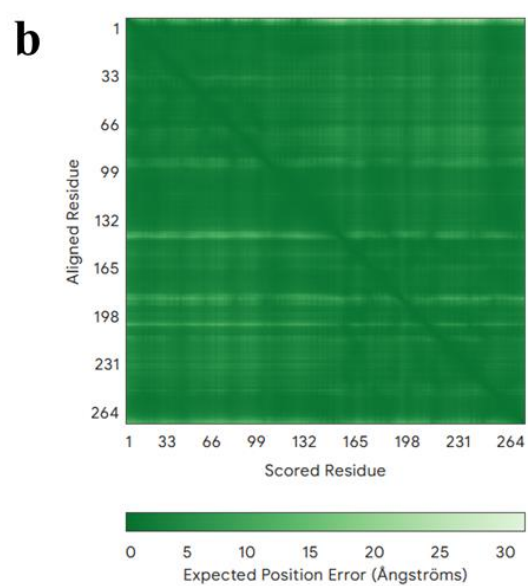

**Predicted aligned error (PAE) map for FhuBCD\_Ea complex (a) and for FhuD\_Ea (b)**

Supplement: S1 Fig — (PDF) [file pone.0326667.s001.pdf]

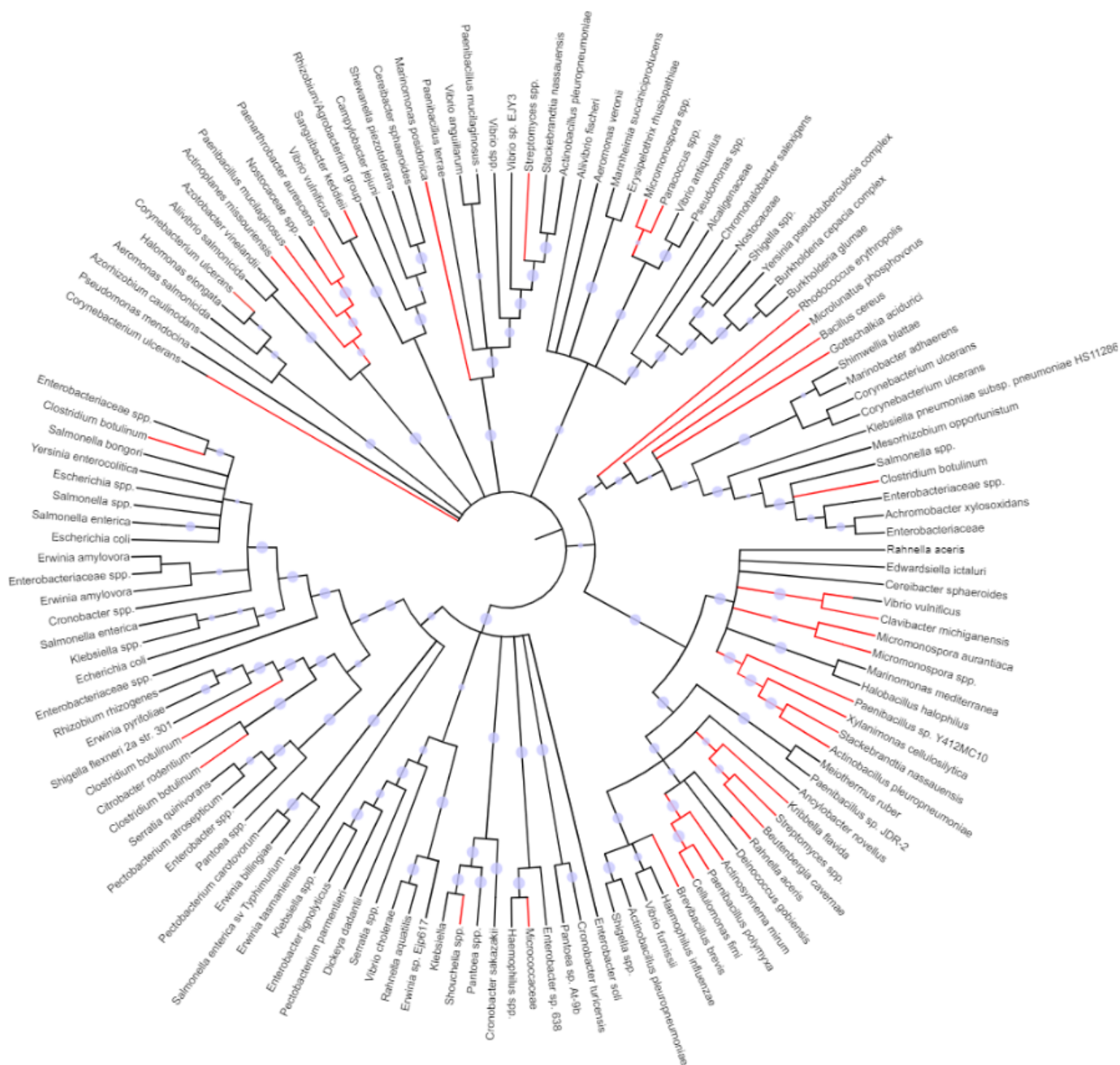

Supplement: S5 Fig — (PDF) [file pone.0326667.s005.pdf]
