# Supplementary material for: Bioinformatics and modelling studies of FhuD, the periplasmic siderophore binding protein from the plant pathogen Erwinia amylovora
Source: PLoS One. 2025 Jul 23;20(7):e0326667. doi: 10.1371/journal.pone.0326667 (PMC12286361; doi:10.1371/journal.pone.0326667)
Supplement: S2 Fig — (PDF) [file pone.0326667.s002.pdf]

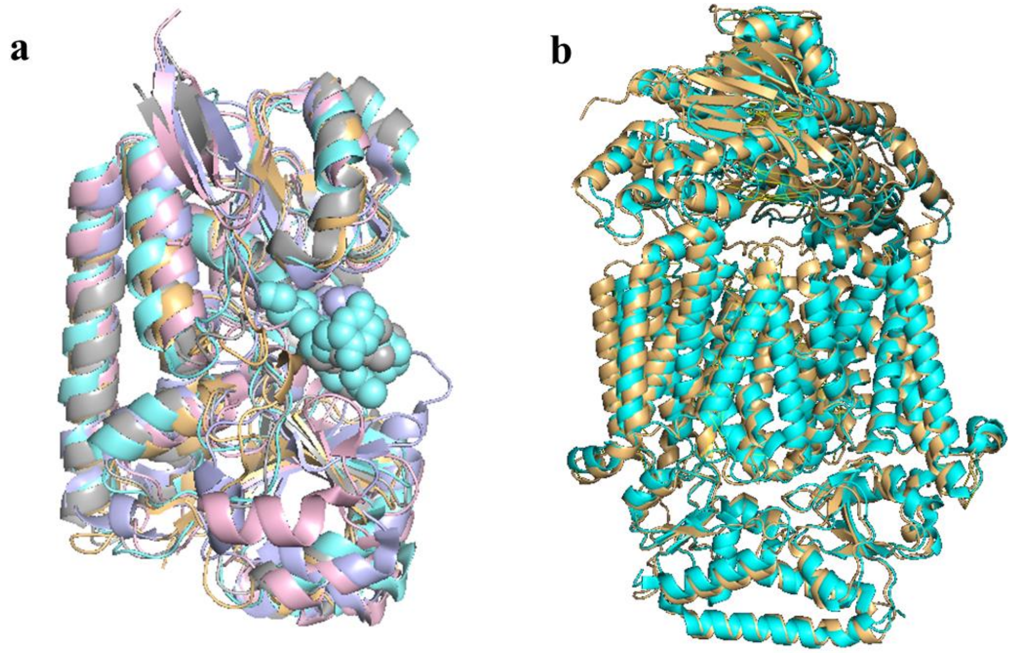


Superimposed structures of FhuD homologs (a) and FhuBCD complexes from *E. amylovora (wheat)* and *E. coli (cyan) (b)*
