# Supplementary material for: Bioinformatics and modelling studies of FhuD, the periplasmic siderophore binding protein from the plant pathogen Erwinia amylovora
Source: PLoS One. 2025 Jul 23;20(7):e0326667. doi: 10.1371/journal.pone.0326667 (PMC12286361; doi:10.1371/journal.pone.0326667)
Supplement: S3 Fig — (PDF) [file pone.0326667.s003.pdf]

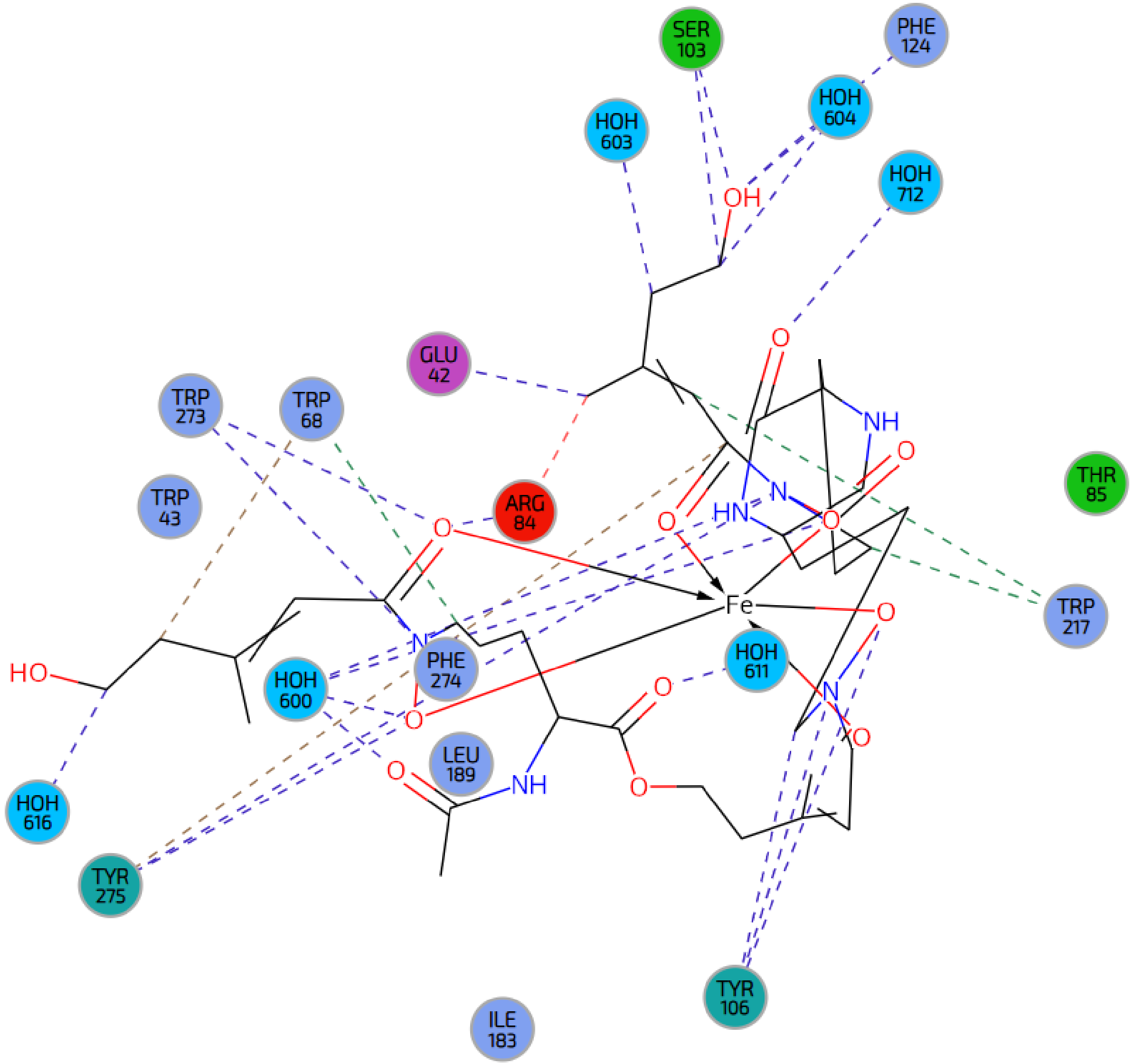

CPO (*E. coli*)

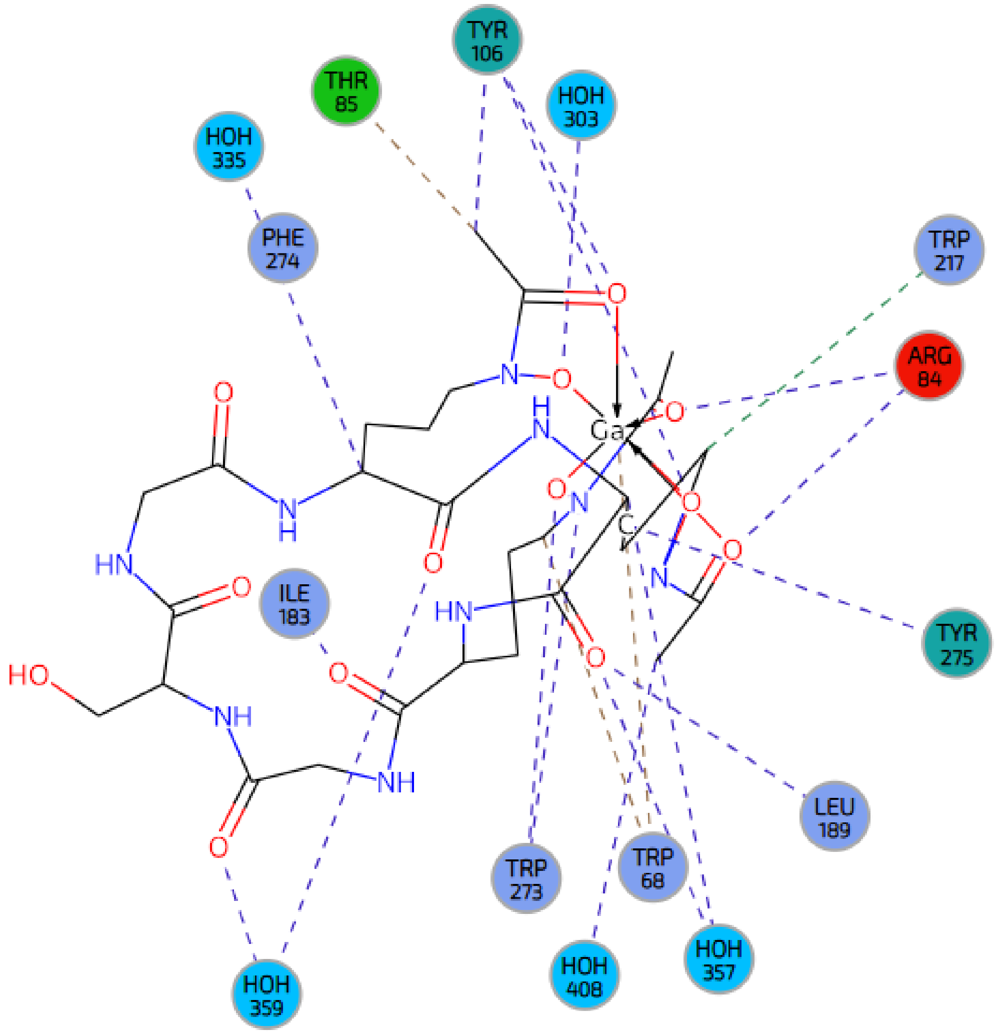

GCR (*E. coli*)

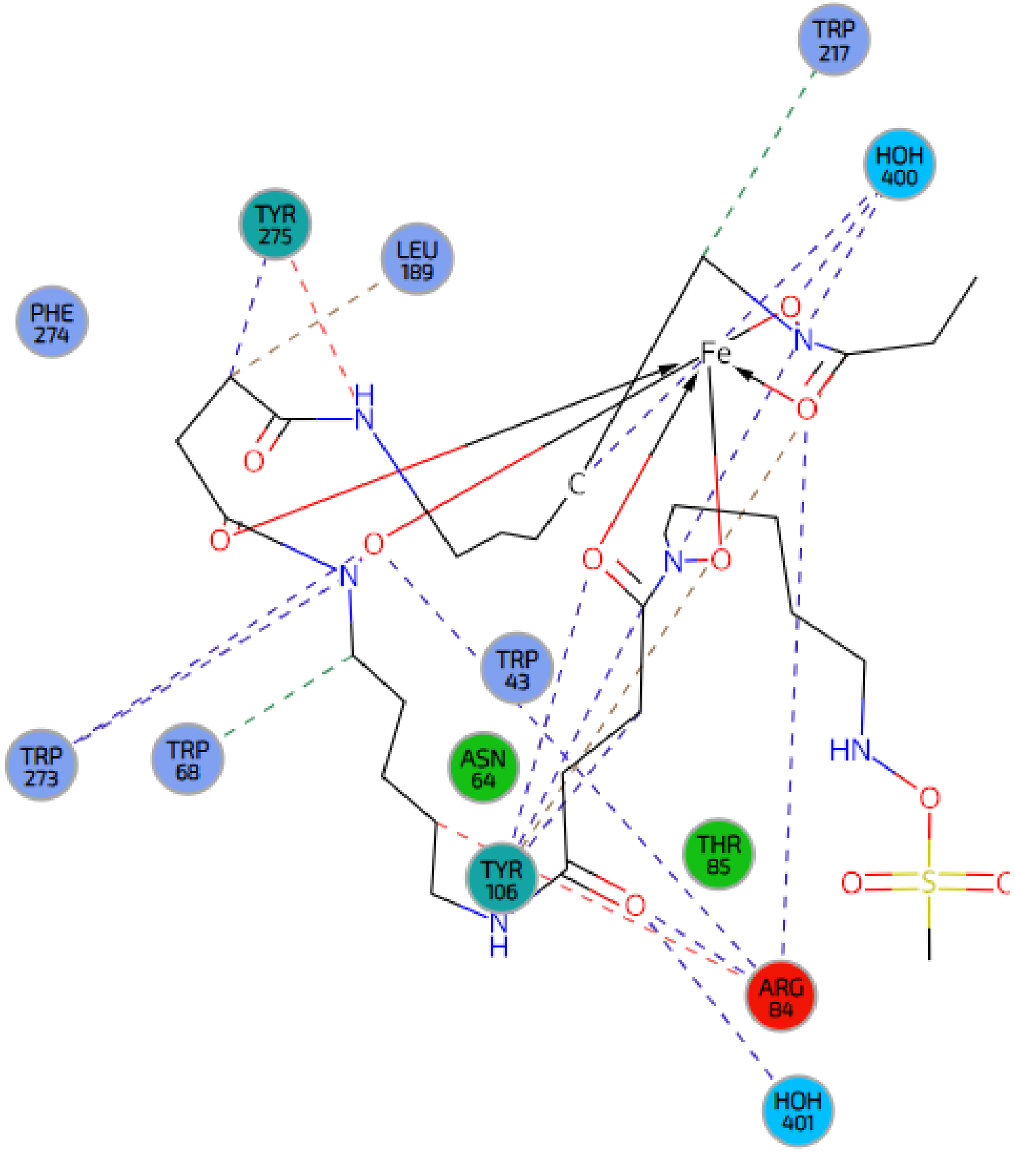

DEF *(E. coli)*

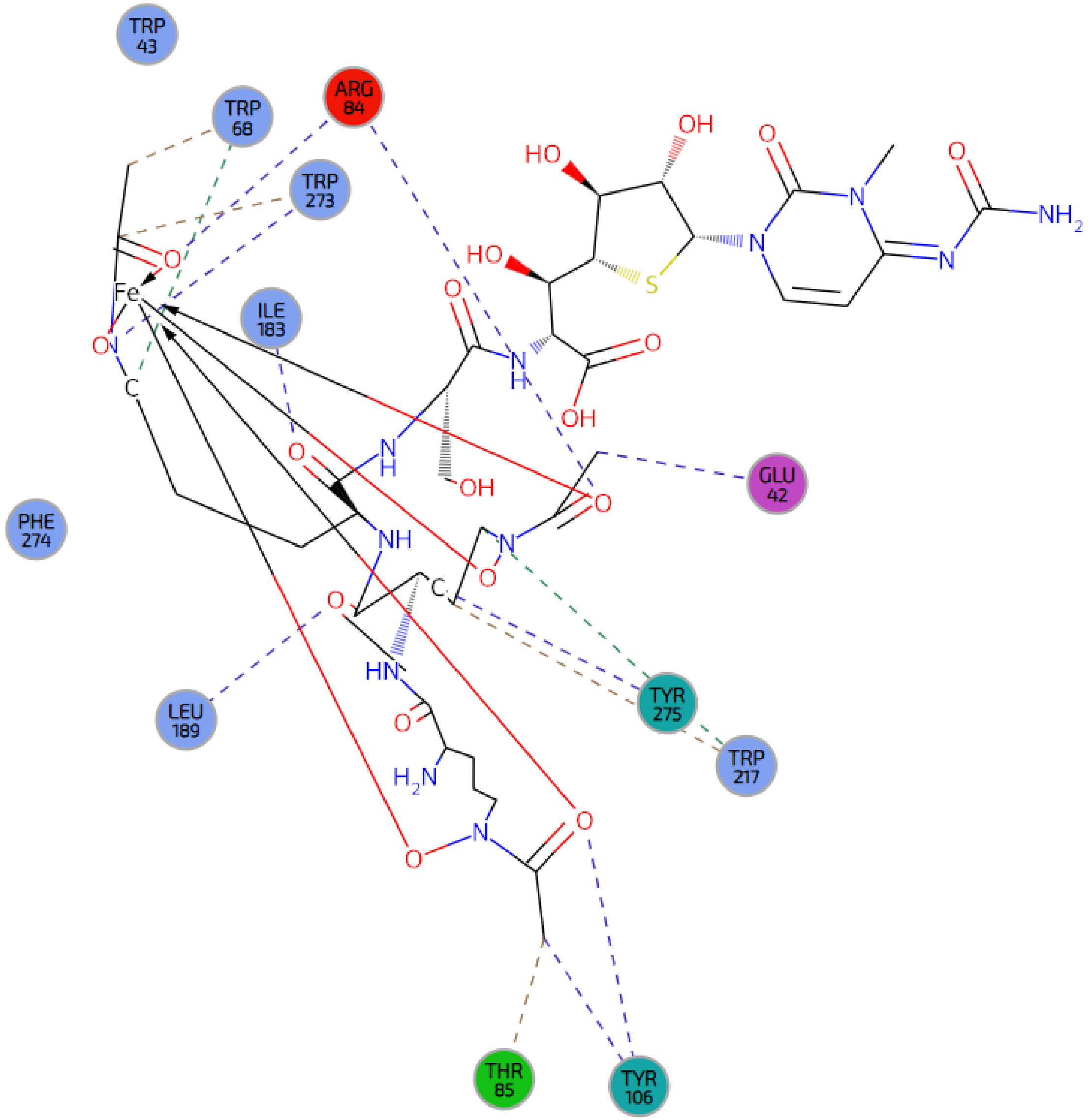

ALB *(E. coli)*

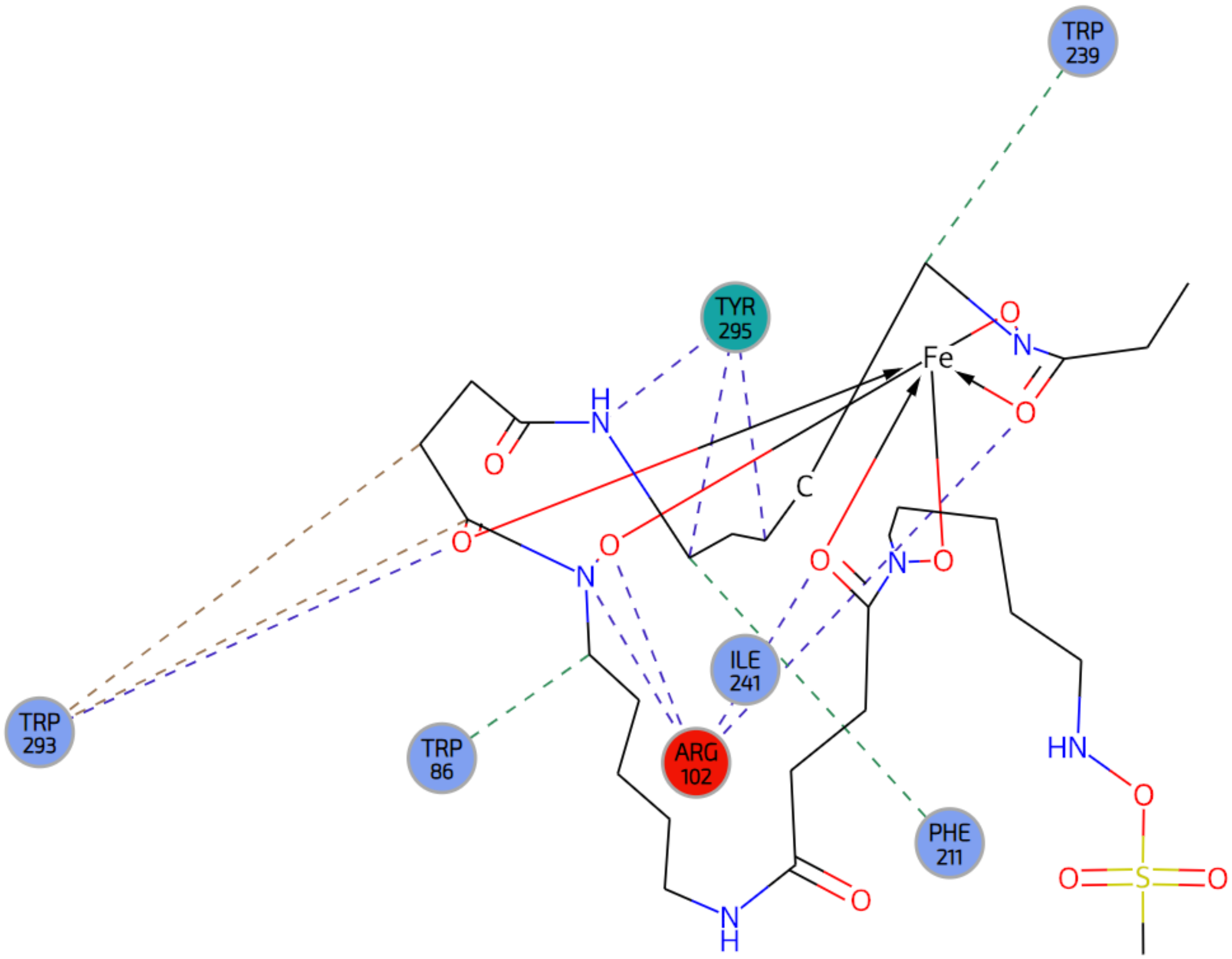

DEF *(V. cholerae)*

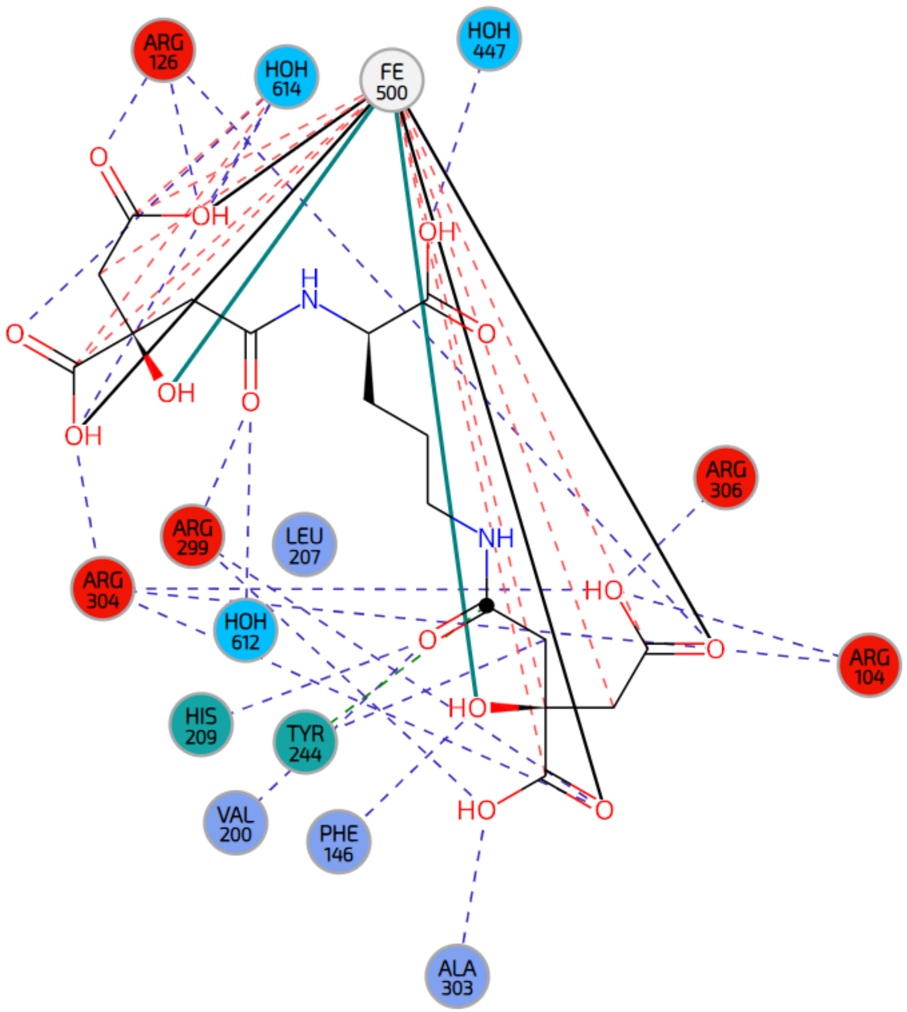

SF8 *(S. aureus subsp. aureus str. Newman)*

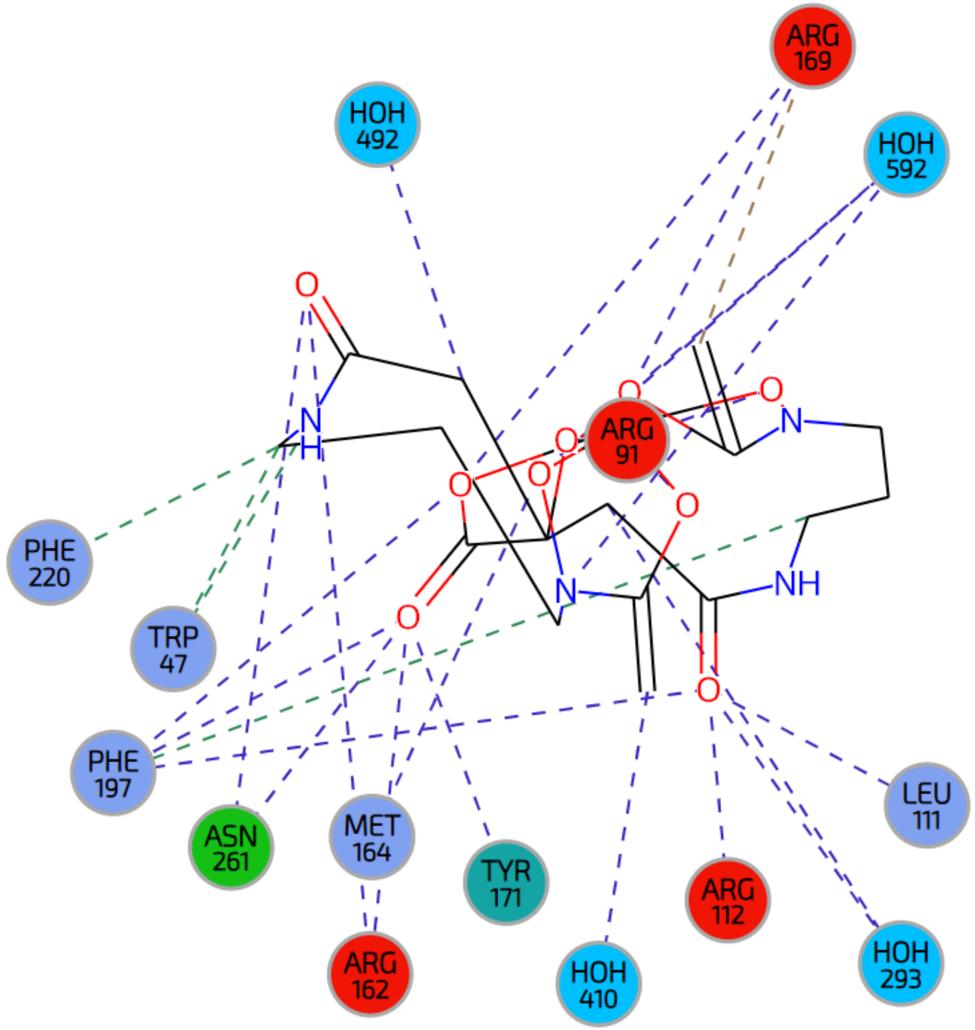
ARG 169
HOH 492
HOH 592
ARG 91
PHE 220
TRP 47
PHE 197
ASN 261
MET 164
TYR 171
ARG 112
LEU 111
ARG 162
HOH 410
HOH 293

SKZ *(B. cereus)*
