# Supplementary material for: Bioinformatics and modelling studies of FhuD, the periplasmic siderophore binding protein from the plant pathogen Erwinia amylovora
Source: PLoS One. 2025 Jul 23;20(7):e0326667. doi: 10.1371/journal.pone.0326667 (PMC12286361; doi:10.1371/journal.pone.0326667)
Supplement: S4 Fig — (PDF) [file pone.0326667.s004.pdf]

```
*Erwinia*                                                    α1
                                         1              10
Erwinia                    .............................MPDLP......RRRLLTALA...........
Cereibacter                .........................MVGGGALSR.........RSFLAA...........
Rhizobium/Agrobacterium    .........................MTRKDRWIS.........PLWGRR...........
Azorhizobium               .............MPEQPSAGTRRNIGRRTILTLM.........RGGLL...........
Microlunatus               ............................................................
Rhodococcus                MTLVLDTQVTDSEWAQIVDRISRRRFFGGAAGVAAALALSACGTSDAESD..........
Beutenbergia               ..................MNRRLFALPAAA....AVLLLAACGTTEAPADDATTPETDAA
Gottschalkia               ..................MKYAKRMILSFSLLLLVSVFITACNSKTVEKDNEISTQNESE
Corynebacterium            ...................MFTRSERSWRYGAALLLSLLPVFAGAASWQ...........
Marinobacter               .................MVSRFTKFAAVFTAAGLALSACSDSGTKDSATS..........
Corynebacterium            .................MVSRFTKFAAVFTAAGLALSACSDSGTKDSATS..........
Corynebacterium            .........................MTRRGALALF.........VLPLV...........
Mesorhizobium              .................MNFKTL.IVCFALIFVTG.........CTPQK...........
Erysipelothrix             ....................MIVHSIKRLGL..........TTLLLCLS...........
Marinomonas                ..............................................MNN...........
Pseudomonas                ..........................MALCL..........ISLLLPLA...........
Aeromonas                  ............................................................
Pseudomonas                .............................MRVLFA.........ALLLT...........
Azotobacter                .....................MNKLIGFIIL.FFISS.....VQAMEIK...........
Aliivibrio                 .....................MYKVISLIMLALYAMS.....SQAIEIK...........
Aliivibrio                 ..MAHTCATVSPRRPAPDSGGSAAGLGCVPALERRRFCLGGVAYLAWAG...........
Alcaligenaceae             ...........................................MKKIII...........
Campylobacter              .........................MVGACGFSR.........RAFLAA...........
Paracoccus                 .................MRPFCNREGLRRSR..........LMTLLTLV...........
Chromohalobacter           .........................................MRILT..A...........
Mannheimia                 .....................MSSTNTRLKH..........FSFALSLS...........
Vibrio                     .....................MKRAGWLLCL..........LAPLGLQA...........
Aeromonas                  .............MSSDGPLDIFR.LSRRNALIAA.........AAMLA...........
Rhizobium                  .............................MMNPT...LITRRRLLIAMA...........
Klebsiella                 .......................MALTLSPLLLSLP.SLVA....AAPK...........
Yersinia                   .............................MRDLY...PLTRRRLLTAMV...........
Enterobacteriaceae         .............................MSGLP...LISRRRLLTAMA...........
Salmonella                 .............................MSGLP...LISRRRLLTAMA...........
Shigella                   ................................MR.....KLLKTLLGMT...........
Shigella                   ...................MSMKIHKALAATAALAVL.LTGCGTTEGPA..SSASNSAG.
Haemophilus                .............................MSDISNFSGITRRRLLQMMA...........
Micrococcaceae             .............................MSDISNFSGITRRRLLQMMA...........
Rahnella                   .....................MRLLTPALLAAAV...LLSACGTTESTS..SEPSASGDA
Rahnella                   .......................MKKRHVFVAASAFMLLAACNS.SNEGNGQ...ENTSK
Actinosynnema              ....................MFRTRVTALVAAAATALLLAACGTTEN.T..AEPAPSASA
Shouchella                 .......................MALTLSPLLLSLP.SLVA....AAPK...........
Micromonospora             ...................MSLRSVRRIRPFLCAALFGTAALAGAQSATP..........
Yersinia                   ...........MKRLIFLPRNTQKHTENIYRIFRGVLCNSVVKTLQAVV...........
Deinococcus                .....................MR.LLRCAAGMVAAAVLLTACGTSEP....GADSEKDDK
Actinobacillus             .....................MRNVRTFILIAFTLAIVLAGCGGGAGTDANTGGGTAASP
Stackebrandtia             .....................MRNVRTFILIAFTLAIVLAGCGGGAGTDANTGGGTAASP
Paenibacillus              ..................MLKRVTF.........VLL.LSLPSMVFATG...........
Paenibacillus              .....................MKKFITMLFTVLAFNALALESVHALEIK...........
Shewanella                 .........................MVGGGALSR.........RSFLAA...........
Vibrio                     ................MCLIFNFIFR.........FFAPLALLFSLFSP...........
Cereibacter                .....................MKQMKWTGLILAAMMVFLAACGGGNNAGNNKQESEQASS
Edwardsiella               .....................MKKIGWLILVLLVVTLTACGNGTDDGGEASEGSGDQSKE
Paenibacillus              ....................MKRLIRLAAAAAT.VAVTVTACGTTEVEEPAAAGSAAPVS
Halobacillus               .....................MKKMLNGLLLFMVFAIVLAGCGSVG..DNSKPEATSGSE
Actinoplanes               .....................MNKVIVLLLSICTFSS......YAFDVT...........
Paenibacillus              ................MRLVRRLLQR.........LAAIAILVGGMLSQ...........
Vibrio                     ....................MR.RHLLTTAAVAVAALSLAACGTTEPSS..DDASAASGK
Vibrio                     ................MCLN.........LVKRWLVAGLLSVFSSVAF............
Streptomyces               ...................MRNLRALPAAALAGTAALLLAACGTTEEAGAAEPSTSADVA
Vibrio                     ..................MLSRVHA.........TTINLALLLFMSSPT...........
Cellulomonas               ....................MLRTRLTLLAAAAASALLIAGCGTTEEPA..AAPSPSDAA
Marinomonas                .....................MKKGFIGFLLLLTFMLTLAGCGATDNAANKGQTATSGSK
Micromonospora             ....................MTERPGPTLRIPAPRRAASRWLAALLTLC...........
Paenibacillus              ....................MLRTRLTLLVAAAASALLIAGCGTTEEPA..AAPSPSDAA
Halomonas                  .............MPT.........LSRR.AMLLG.........GLATS...........
Micromonospora             ...........................MKRLISGFLFILASLTAAQ..............
Ancylobacter               ...................MRRITALAATL.....LLALTACGSSETPE.....PAAAEA
Meiothermus                ..................MSLRRTAPLASSAALLLALSLSACGTTDPGAAAAA.PAGTAD
Kribbella                  .................MRTSRLLALPAALLT..AALTLTACGTTEPAG.SETTDGAPAA
Xylanimonas                .....................MRSMKGLVFIVLIFAVVLAGCGGGAASNEGSGSTAAQNS
Sanguibacter               .........................MPRPDPQRRRWLARGGAFGLLGAL...........
Paenibacillus              .....................MRLIKLPVMLVFMSAMLLLAACGNAGSTAQSGSNQAATA
Burkholderia               ...........MKQLIFLPRNTQKYTENIYRIFRGVPCNSVVKTLQAVV...........
Brevibacillus              ..................MITRRRTLAMTALAAATALTLTACGTTEEAS..TGAGTTP..
Actinobacillus             ...................MSMKIHKALAATAALAVL.LTGCGTTEGPA..SSASNSAG.
Clavibacter                ....................MIKFTRRTFLTAATASALTAACNRSP..............
Paenarthrobacter           .....................MKKIILMLSAALAFNS......YALDIT...........
Nostocaceae                .....................MKKIILMLSAALAFNS......YALDIT...........
Vibrio                     ....................MSKFTRRAFLTTATASALTVACGQRE..............
Vibrio                     .......................MSLRAANPRRRQLLVA......LTAL...........
Nostocaceae                ................................MR.....KLLKTLLGMT...........
Burkholderia               ...........MKQLIFLPRNTQKYTENIYRIFRGVPCNSVVKVLQAVV...........
Haemophilus                ....................MKNQHLTWPVLAAAAALALTACGTTEAPK..KESAKDS..
Actinobacillus             ................MCLMSLWNVRLRNICRPKAVFGWLAALCLFALP...........
Streptomyces               ................MCLMSLWNVRLRNICQPKAVFGWLAALCLFALP...........
Vibrio                     ...........................MPDSSDFHH.DAVRRRLLTAMA...........
Vibrio                     .............................MRDLY...PLTRRRLLTAMA...........
Vibrio                     .............................MPDLF......RRRLMMAMA...........
Serratia                   .............................MLDSM...LMSRRRLLTAMA...........
Salmonella                 .............................MRDILST.PFSRRRLMTAMA...........
Pantoea                    .............................MRDP....MISRRRLLTAMA...........
Enterobacter               ........................MMPEFFSSPRSP.DPLRRRLLTALL...........
Cronobacter                ........................MMPECFSSPSSP.DPLRRRLLTALL...........
Enterobacter               .............................MSDISSISGITRRRLLQMMA...........
Pectobacterium             .............................MLNST...LLSRRRLLTAMA...........
Pectobacterium             .............................MRDILST.PFSRRRLMTAMA...........
Rahnella                   .............................MPDLF......RRRLMMAMA...........
Klebsiella                 .............................MPDLP......RRRLLTALA...........
Enterobacter               .............................MLDS....MISRRRLLTAMA...........
Cronobacter                ...........................MPASSDFQH.DPVRRRLLTAMA...........
Pantoea                    .....................MKMHLPIKHIHLTPP.DLTRRRLLTALA...........
Erwinia                    .............................MLDIS......RRRLLAALA...........
Klebsiella                 .............................MGNSP...VISRRRLLMALA...........
```

**Enterobacter**	........................MLNKMMGNPNEP.DALRRRLLAAML...........
**Serratia**	.............................MPDLI......RRRLLMAMA...........
**Yersinia**	.............................MSGLH...HLTRRRLLTAMA...........
**Pantoea**	........................MMPEFFSSPHYP.DPLRRRLLTALL...........
**Enterobacter**	.............................MPDLP......RRRLLTALA...........
**Dickeya**	.............................MPDLM......RRRLLTALA...........
**Erwinia**	.............................MRDVLST.PFSRRRLMTAMA...........
**Klebsiella**	.....................MKMHLPIKHIHLTPP.DLTRRRLLTALA...........
**Citrobacter**	.............................MENLT...FITRRRLLTAMA...........
**Pectobacterium**	.............................MPDLP......RRRLLTALA...........
**Erwinia**	.............................MMNPT...LITRRRLLIAMA...........
**Erwinia**	.............................MNQPVSA.ALSRRRLLTLMA...........
**Cronobacter**	.............................MSGLP...LISRRRLLTAMA...........
**Yersinia**	.............................MSGLP...LISRRRLLTAMA...........
**Klebsiella**	.............................MSGLP...LISRRRLLTAMA...........
**Klebsiella**	.............................MRDLY...PLTRRRLLTAMV...........
**Shimwellia**	.................MVSRFTKFAAVFTAAGLALSACSDSGTKDSATS..........
**Escherichia**	................MCLMSLWNVRLRNICRPKAVFGWLAALCLFALP...........
**Enterobacteriaceae**	.............................MRELY...PLTRRRLLTAMA...........
**Enterobacteriaceae**	.............................MRDLY...PLTRRRLLTAMA...........
**Escherichia**	.............................MRDLQ...PLTRRRLLTAMA...........
**Escherichia**	.............................MSGLP...LISRRRLLTAMA...........
**Escherichia**	.............................MSGLP...LISRRRLLTAMA...........
**Salmonella**	.............................MSGLP...LISRRRLLTAMA...........
**Salmonella**	.............................MSGLP...LISRRRLLTAMA...........
**Salmonella**	.............................MMNPT...LITRRRLLIAMA...........
**Salmonella**	.............................MSGLP...LISRRRLLTAMA...........
**Salmonella**	.................MNKKIWTIVGILIVFIVGGTLFFKPKEKKEDV...........
**Salmonella**	.............................MSGLP...LISRRRLLTAMA...........
**Enterobacteriaceae**	.................MNKKVWTIVGILIVFIVGGTLFLKPKENKKEE...........
**Enterobacteriaceae**	.............................MSGLP...LISRRRLLTAMA...........
**Enterobacteriaceae**	.................MNKKVWTIVGILIVFIVGGTLFFKSKEKKEDV...........
**Escherichia**	.............................MRDLY...PLTRRRLLTAMA...........
**Klebsiella**	...................MKKQTQAGRVRRWAVQAGAALVLGAATAVC...........
**Clostridium**	.............................MRDLY...PLTRRRLLTAMA...........
**Clostridium**	.................MNKKVWTIVGILIVFIVGGTLFLKSKEKKEDV...........
**Clostridium**	.............................MENLT...FITRRRLLTAMA...........
**Achromobacter**	.................MNKKVWTIVGILIVFIVGGTLFLKSKEKKEDV...........
**Clostridium**	.............................MMNPT...LITRRRLLTAMA...........
**Clostridium**	.............................MSGLP...LISRRRLLTAMA...........
**Shigella**	.............................MMNPT...LITRRRLLIAMA...........
**Bacillus**	............................MKKKLTILFSIMCILVLAACG...........
consensus>70	....

```
                                                            β1             α2
*Erwinia*                           TT                     ──►        ℓℓℓℓℓℓℓℓℓℓ
                                    20         30                     40     50
Erwinia                     .............FSPLLARLPGVAH..AAGE.PRVIALE......WLPLELLMALGVTP
Cereibacter                 .............ASLALAAASSLR..AAPPVAPRLAAID......WAMLETAIALGHMP
Rhizobium/Agrobacterium     .............EFLSLLAASALAGKARAGVTPRIAAID......WAMLETSVALGVMP
Azorhizobium                .............AAPFGVSAGPAPADATP.PLRRLVALD......YGLAETLLLLGLPP
Microlunatus                ...........................MYTSDLDYAITLG.......LPLAPVQSIREGS
Rhodococcus                 ............SASDTLPYTWGEFSGDVPRDPKRVVVLDGRVDLEFAMMMDYPIVGSGN
Beutenbergia                ............ASGPVTIVDDRGEEITLDAPAVDVVSLE......WGLTEHLLSLGVTP
Gottschalkia                .............KSKTRKYKDYMGHEVDIPASPKRVIFH......GENFGDLLAIGVDA
Corynebacterium             .............HEQGTLTLNKTPER........VIALN......WAATEALLLLGVTP
Marinobacter                ..........AQSSANTVTVQDNYGTVEIKTPVERVVATD......NRTFQVLDQWGVTP
Corynebacterium             ..........AQSSANTVTVQNNYGTVEIKTPVERVVATD......NRTFQVLDQWGVTP
Corynebacterium             .............ATMVRPADAK.........PLRVVCLD......DGLAETLLMLGVTP
Mesorhizobium               ............TKDTTLSITHKLGTTEVNENPQNVFVFD......MGIIDMMESYDLPI
Erysipelothrix              .............SWTLN.....AEPQ.......RLVSID......WSHTETLLGLGVTP
Marinomonas                 .............LLRCVPLLLCLLAGLAQAAEPRVVALS......WEAAEHLLKLDITP
Pseudomonas                 .............AQADTSPISSVPLP.......RIATVD......WTIAETLLALGVTP
Aeromonas                   ..............MAIGGLALGCGLPLRAEPLQRVVAIN......WGAAETLLTLGVEP
Pseudomonas                 .............LALPAPAAPP..........QRIAAID......WGLAETLLGLGVTP
Azotobacter                 .............HELGTTQFTETPKK........VVVLD......WALTETVLSLGIVP
Aliivibrio                  .............HELGSTEIEVTPQK........IVALD......WVLAETVLSLGVEP
Aliivibrio                  .............TVPLGALARTDSAQTLGTRGARIACTD......WAAAESLALLGCMP
Alcaligenaceae              .............LFLILLDLTQSKELK.......IISLD......WAIAETMMMLNYQP
Campylobacter               .............A..CAVAGRALP..AFAADGLRLAAVD......WAMAETAMALGHPP
Paracoccus                  .............LLV.WSHVANAETP.......RIATVD......WTIAETLLALGVTP
Chromohalobacter            .............LLLFFSLSVNAQIK........IATLD......WTVAETLIALNNAP
Mannheimia                  .............ALLVSSFQAVAEASP......RVMSVD......WTQTETMLALGVVP
Vibrio                      .............SPASQSAITDTARPSEHTQVLRIATVD......WTIAETLLALGVTP
Aeromonas                   .............APRIGCAASPMSG........PIVSLD......YGLASTLLALGVTP
Rhizobium                   .............LSPLLWQMR.GAQ.AADVDPQRVVALE......WLPAELLLALGVTP
Klebsiella                  .............SDQPLLNIDRVIDIQRDIDTKRVVALE......WLPVELLLALGVTP
Yersinia                    .............LSPLLWQMN.TAQ.AAAIDPRRIVALE......WLPVELLLALGITP
Enterobacteriaceae          .............LSPLLWQMN.TAH.AAAIDPNRIVALE......WLPVELLLALGIVP
Salmonella                  .............LSPLLWQMN.TAH.AAAIDPNRIVALE......WLPVELLLALGIVP
Shigella                    .............ALLAHSVLFAASHNG.......YTTVD......WVAAETLIALGEPP
Shigella                    ............AGEAITVTDARGTEVKLDGPAKRVVGTE......WNVVENLTTLGVMP
Haemophilus                 .............LSPLLTSLP..ALSASRPDLSRIIALE......WLPAELLIALGVMP
Micrococcaceae              .............LSPLLTSVP..ALSASRPDLSRIIALE......WLPAELLIALGVMP
Rahnella                    ............G.GAITLTDARGKTVELKAPAKKVVSLE......WAETEMVLTLGVTP
Rahnella                    DGNS.........DTGSITVTDTQGEQTFTEPAKRVVALD......WTYVENLLALGVQP
Actinosynnema               ............AGGPVTVTDSRGKEIKLDSPATKVVGLE......WGEVEMLVSLGVMP
Shouchella                  .............SDQPLLNIDRVIGIQRDIDTKRVVALE......WLPVELLLALGVTP
Micromonospora              .............ACAGQLVRDVTGQVCIPKAPKRIVTIE......WTYSENLLALGIQP
Yersinia                    .............FLLFFVISCGVKAKS.......FATLD......WTVAETLIALGEKP
Deinococcus                 ............G.GPVTVTDARGKEVKLKSPADRVVSLE......WAETEMLVSLGVMP
Actinobacillus              N...........SAAQTRTIKHALGETTITGVPKRIVALE......WLYAEDVLALGIQP
Stackebrandtia              N...........SAAQTRTIKHALGETTITGVPKRIVALE......WLYAEDVLALGIQP
Paenibacillus               .............TVVAKTVIDSRGEQQLTATPKRVATLN......WDIAEQVLALGVIP
Paenibacillus               .............HEMGSVSFDATPKK........VVALD......WALTETVLSLGVEL
Shewanella                  .............ASLALAAASSLR..AAPPVAPRLAAID......WAMLETAIALGHMP
Vibrio                      .............SAQALTVSDSRGTHQLPAVPTRAVVLD......WDLLEQTIELGVTP
Cereibacter                 NANANASTNTAADETAERTIKHALGETKITGTPKKVVVLE......WTYAEDLIALGVQP
Edwardsiella                AKEEEK......DEQRTINIQDAMGEKTIEGTPKNIVVLE......WTYAEDLQALGMEP
Paenibacillus               QTCANDT..TTTATGPVSMTDGVGRTVKLDKPAQRVAVLE......WQQTEDLLTLCLNP
Halobacillus                QTTK.........AAGPVTVKDDHGEVKLDKPAERVVVLE......WAYTEDLIALGVQP
Actinoplanes                .............HEMGVASFESPPQN........VIALD......WGLAETVLSLGVQP
Paenibacillus               .............AHAAITISDDYGSHSFAQTPQRVVALN......WDILEQVLALDVEP
Vibrio                      ............PAEKITLTDAKGTKVTLDGPATKVVATE......WNVVEDLVALGVDP
Vibrio                      ..............SAPLTVHDSRGEQTLQQRPQRVVVLN......WDLLEQVIELGITP
Streptomyces                ............G.GPVTITDDRGEEVTLDAPATDVVSLE......WGLTENLLTLGVTP
Vibrio                      .............SLFAVTMMDQYGESVFESAPKRIAALS......WELAEDLVELGVTP
Cellulomonas                ............STGPVTVTDSRGKTITLKSPATKVVGLE......WGEVEMLVGLGVMP
Marinomonas                 PTEA.........ATGPVTVKHNRGELTLDKPAQRVVVLE......WTFTEDLIALGVQP
Micromonospora              .............CLVGASTALAADPP.......RIATLD......WTLAETLQALGTPP
Paenibacillus               ............STGPVTVTDSRGKTITLKSPATKVVGLE......WGEVEMLVGLGVMP
Halomonas                   .............LALPRPAGAQAGRDAPAFPPSRIVSMD......FGLAETLIEMGLPP
Micromonospora              .............ACTGRLVQHALGETCVVGTPKRVVALE......WTYAEYLLALGLQP
Ancylobacter                ............SAEPVSLTDARNKKIDLPAPATKVVGLE......WGVVEGLITLGVMP
Meiothermus                 GECAD....VATSTGPVSLTDSFGRTVELDKPAERVAVLE......WQQIEDTLTLCVTP
Kribbella                   ............SSGPVTYTDERGE.HTLDAPATAVVSLE......WGLTENLLALGAPI
Xylanimonas                 DQGAAQGGDKTEAAPETRTIKHAMGETKITGVPKRVVALE......WLYAEDVLALGVQP
Sanguibacter                .............AWCAPLRAAAPAAAAPARFPTRVVSMN......WELTETLLALGVVP
Paenibacillus               QPDAAKP.....AEEQVRTVKHMMGESTIKGTPKRIVALE......WSSAEHLLALGIQP
Burkholderia                .............FLLFFVISCGVKAKS.......FATLD......WTVAETLIALGEKP
Brevibacillus               ............AGEQITLTDGTGAEVTLDGPATKVVGTE......WNVVENLVSLGVDP
Actinobacillus              ............AGEAITVTDARGTEVKLDGPAKRVVGTE......WNVVENLTTLGVMP
Clavibacter                 ........................RPQAASKTATRVVALE......WVYAENLLALGIQP
Paenarthrobacter            .............HEMGTTSFETTPKK........VVALD......WVLTETVLSLGIEL
Nostocaceae                 .............HEMGTTSFETTPKK........VVALD......WVLTETVLSLGIEL
Vibrio                      ........................QPQTSNQTATRVIALE......WVYAENLLALGIQP
Vibrio                      .............AGLGP.QFSAPCAAAVARSPHRIVVLN......WELTETLLALGRAP
Nostocaceae                 .............ALLAHSVLFAASHNG.......YTTVD......WAAAETLIALGEPP
Burkholderia                .............FLLFFVISCGVKAKS.......FATLD......WTVAETLIALGEKP
Haemophilus                 ...............AVTITDARGKKITLDGPAERVVGTE......WNVVESLVTLGVQP
Actinobacillus              .............VQAQIVLTDSQGTHNFAEVPQRVVVLN......WDLLEQVLELGIQP
Streptomyces                .............VQAQIVLTDSQGTHTFAEVPQRVVVLN......WDLLEQVLELGIQP
Vibrio                      .............LSPLLFSLPGQAAATP.PDLARIVALE......WLPIELLLALGVTP
Vibrio                      .............LSPLLWQMN.TAQ.AAAIDPRRIVALE......WLPVELLLALGITP
Vibrio                      .............LSPLMSPFSLQAA..QPDP.DRIIALE......WLPTELLLALGVKP
Serratia                    .............LSPLLLKMN.TAR.AAAVDPHRIVALE......WLPVELMMALGVTP
Salmonella                  .............LSPLMWHMA.KAR.AASVDLKRIVALE......WLPVEQLIALGVVP
Pantoea                     .............LSPLLLKMG.TAH..AAINPHRIVALE......WLPVELLLALGITP
Enterobacter                .............LSPLVYSAASRGGTSAFPDLNRIVALE......WLPVELLLALGITP
Cronobacter                 .............LSPLVYSAASRGATATFPDLHRIVALE......WLPVELLLALGITP
Enterobacter                .............LSPLLTSLP..ALSASRPDLSRIVTLE......WLPTELLIALGVMP
Pectobacterium              .............LSPLLLKMN.LARAAAAVDPHRIVALE......WLPVELMMALGVTP
Pectobacterium              .............LSPVMWHMA.KAR.AASVDLKRIVALE......WLPVEQLIALGVMP
Rahnella                    .............LSPLMSPFSLQAA..QPDP.ARIIALE......WLPTELLLALGVKP
Klebsiella                  .............FSPLLARLPGVAH..AASA.PRIIALE......WLPLELLMALGVTP
Enterobacter                .............LSPLLLKMN.TAR.AAAIDPHRIVALE......WLPVELMLALGVTP
Cronobacter                 .............LSPLLFSLPGRAAVTP.PDLTRIVALE......WLPIELLLALGVTP
Pantoea                     .............LSPLLYSLSGWAVNPPKIDSQRVVALE......WLPVELLLALGVMP
Erwinia                     .............LSPLMQLAPLHAA..LPDT.RRILALE......WLPTELLMALGVAP
Klebsiella                  .............LSPLLWQMK.NAQ.AGAINPHKIVALE......WLPVELLLALGVTP
```

```
Enterobacter            .............VAPWFSPFATR...AAAVDSQRVVALE......WLPVELLMALGVTP
Serratia                .............LSPLLPALRTQAA..ASVDTQRIISLE......WRPTELLMALGVPP
Yersinia                .............LSPLLWQMR.PAR.AASIDLQRIVALE......WLPVELLLALGVTP
Pantoea                 .............LSPLVYSAASRGAIATPPDVNRIVALE......WLPVELLLALGLTP
Enterobacter            .............FSPLLARLPGVAH..AASA.PRIIALE......WLPLELLMALGVTP
Dickeya                 .............CSPLLARLPGVAH..AASA.PRIIALE......WLPLELLMALGVMP
Erwinia                 .............LSPLMWHMA.KAR.AASVDLKRIVALE......WLPVEQLIALGVMP
Klebsiella              .............LSPLLYSLSGWAVNPPKIDSQRVVALE......WLPVELLLALGVTP
Citrobacter             .............LSPLLWQMR.GAR.AAEVDPQRIVALE......WLPVELLLALGVTP
Pectobacterium          .............FSPLLARLPGVAH..AAGE.PRVIALE......WLPLELLMALGVTP
Erwinia                 .............LSPLLWQMR.GAQ.AADVDPQRVVALE......WLPAELLLALGVTP
Erwinia                 .............LSPLLWRSA.SLH.AAPVDSQRVVALE......WLPVELLIALGVTP
Cronobacter             .............LSPLLWQMN.TAH.AAAIDPNRIVALE......WLPVELLLALGIVP
Yersinia                .............LSPLLWQMN.TAH.AAAIDPNRIVALE......WLPVELLLALGIVP
Klebsiella              .............LSPLLWQMN.TAH.AAAIDPNRIVALE......WLPVELLLALGIVP
Klebsiella              .............LSPLLWQMN.TAQ.AAAIDPRRIVALE......WLPVELLLALGITP
Shimwellia              ..........AQSSANTVTVQDNYGTVEIKTPVERVVATD......NRTFQVLDQWGVTP
Escherichia             .............VQAQIVLTDSQGTHTFAEVPQRVVVLN......WDLLEQVLELGIQP
Enterobacteriaceae      .............LSPLLWQMN.TAQ.AAAIDPRRIVALE......WLPVELLLALGITP
Enterobacteriaceae      .............LSPLLWQMN.TAQ.AAAIDPRRIVALE......WLPVELLLALGITP
Escherichia             .............LSPLLWRMN.TAQ.AAAIDPHRIVALE......WLPVELLLALGITP
Escherichia             .............LSPLLWQMN.TAH.AAAIDPNRIVALE......WLPVELLLALGIVP
Escherichia             .............LSPLLWQMN.TAH.AAAIDPNRIVALE......WLPVELLLALGIVP
Salmonella              .............LSPLLWQMN.TAH.AAAIDPNRIVALE......WLPVELLLALGIVP
Salmonella              .............LSPLLWQMN.TAH.AAAIDPNRIVALE......WLPVELLLALGIVP
Salmonella              .............LSPLLWQMR.GAQ.AADVDPQRVVALE......WLPAELLLALGVTP
Salmonella              .............LSPLLWQMN.TAH.AAAIDPNRIVALE......WLPVELLLALGIVP
Salmonella              ............ATSVDMKITHKLGEATLKKNPKKVVVFD......YGTLDSLDKMGIEI
Salmonella              .............LSPLLWQMN.TAH.AAAIDPNRIVALE......WLPVELLLALGIVP
Enterobacteriaceae      ............ASSVDIKITHKLGEATLKKNPKKVVVFD......YGTLDSLDKMGIEI
Enterobacteriaceae      .............LSPFLWQMN.TAH.AAVIDPNRIVALE......WLPVELLLALGIVP
Enterobacteriaceae      ............ATSVDMKITHKLGEATLKKNPKKVVVFD......YGTLDSLDKMGIEI
Escherichia             .............LSPLLWQMN.TAQ.AAAIDPRRIVALE......WLPVELLLALGITP
Klebsiella              ............AAQATVPVKHARGETAVPANPAKTVVMD......LAVLDTLHALGVDA
Clostridium             .............LSPLLWQMN.TAQ.AAAIDPRRIVALE......WLPVELLLALGITP
Clostridium             ............ATSVDMKITHKLGEATLKKNPKKVVVFD......YGTLDSLDKMGIEI
Clostridium             .............LSPLLWQMR.GAR.AAEVDPQRIVALE......WLPVELLLALGVTP
Achromobacter           ............ATSVDMKITHKLGEATLKKNPKKVVVFD......YGTLDSLDKMGIEI
Clostridium             .............LSPLLWQMR.GAQ.AANIDPQRVVALE......WLPAELLLALGVTP
Clostridium             .............LSPLLWQMN.TAH.AAAIDPNRIVALE......WLPVELLLALGIVP
Shigella                .............LSPLLWQMR.GAQ.AADVDPQRVVALE......WLPAELLLALGVTP
Bacillus                ...................QTKSNKEVTKKGNDPKIASMS......IHLTNNLLALGIKP
consensus>70            ..................................riv.le......w...e.l..lgv.p
```

```
                            β2    α3                  TT β3         α4                 β4 TT
Erwinia                                60        70        80        90        100
Erwinia                     LGAAELYNYRLWV..GKPELPPSVVDVGLRSEPNLELITQMQPS......LILFSQGYGP
Cereibacter                 VAACELIRFRKDA..PEPPVPEGVVDLGLRGAPNFELLQLVRPD......LILTSPYYTR
Rhizobium/Agrobacterium     VAATELIQFRSGA..VEPDIPETVADLGLRGAPNFELLQLTRSE......LILISPFYTR
Azorhizobium                VGLVGAQDWNRWV..GEPALPAGVVNLGSSREPNLELLQQLAPD......AILSTPYLAG
Microlunatus                IGFPDFFPQEPLQ........GIEPLVNFPEFEYEKIAAAEPD......VIINGLGYEG
Rhodococcus                 FWFPDAKAGFQFP..GRTIEDASFVNVAGDFSTNFEALLALEPD....LIVITAIGYTSD
Beutenbergia                VGNADNAGYEAWD..TIVPLPEGVTDVGGRGEPSVDSIVGLDAD......LVVTTTDLPE
Gottschalkia                VGTGNVWIKDHIY....EDRAKDVEDVG..FPVNLEKVLELKPD......LFVFAGSDEK
Corynebacterium             IGVADRDGYNVWV..REPELPEGVANIGTRVAPSLEAIAELKPD......LIVTSSEMAP
Marinobacter                VAVP.KPIAPSTV..PNFLNNDQIVDLGTHREPNLEAMVATDPD......LIISGQRFSQ
Corynebacterium             VAVP.KPIVPSTV..PNFLNNDQIVDLGTHREPNLEAMVATDPD......LIISGQRFSQ
Corynebacterium             VAVADREVWEKWV..VEPPLPPQVADIGTILEPNLEFLQQLRPD......VILSIPYLDG
Mesorhizobium               SGVP.TASLTTTL..KSKLD.PNLTDIGTLFEPNYERISEAHPD......LIVISGRSAK
Erysipelothrix              VGATQKTAYNAWV..KEPSIPIESVDIGLRTQPNLERLAELNPD......HIFVSPAYQY
Marinomonas                 VAVADAEQYRAQV..ARPPLPAQVPSAGSRRAPDFDKLTALKPE......LIVIGERQEA
Pseudomonas                 LAVGDAGPYQAWV..GEPRLPAGVVDIGLRTQPNRELLAELKPD......RILISPLAAP
Aeromonas                   LAISDVGYYRRRM..PGSPLPQTVRDIGPYWEPNLELLHQLNPQ......LILSDALPNS
Pseudomonas                 VAVAQLDGYRRWV..GEPALPAGMVDLGLRSEPNLELLAELRPE......LILIGPPFEN
Azotobacter                 LGAADVEGYQTWV..MEPELNRSVMDVGSRREPNLELMTELNPD......VILINQHMSA
Aliivibrio                  LGVADAKGYQAWV..MAPKLPSNVLDVGSRREPNLELLTELNPD......VILISQHMSA
Aliivibrio                  IAVPELAVYRLWL..PEPPLPANVADLGSRSEPNLELLAALAPE......RIVVSSWQAG
Alcaligenaceae              LAVGDKRIYNVWV..KEPVLPNEIKDVGLRVQPNIEYIINLKPD......LIITSSLFSI
Campylobacter               AALAELIGFRDAA..VEPP.PQGTIDLGLRGAPNLEALSLVAPD......LILSSSYYSA
Paracoccus                  VGVAQTDAYREWV..GAPTLPAEVADIGLRAQPNRELLAQLSPD......RILISPMFST
Chromohalobacter            VAVGDKASYKIWV..GKPALAENTQDLGLRLQPNKESLARLSVD......RFINSDFFAS
Mannheimia                  VGVAQGQDYDAWV..KSPPLPPQTKDVGLRTQPNIERIYELRPE......RIFIAPYFSS
Vibrio                      LAVGDVSAYRAWV..GEPLLPADVVDIGLRAQPNRELLAELKPD......RILISPLAAP
Aeromonas                   AAIVSLVDWDKWV..VEPKMPSGVVDLGTAWEINLEILASLKPA......LILTTPYLAA
Rhizobium                   YGVADIPNYRLWV..NEPALPDSVIDVGLRTEPNLELLTQMKPS......FIVWSAGYGP
Klebsiella                  FGVADIHNYRLWV..GEPALPADVINVGQRTEPNLELLQQMAPS......LILLSQGYGP
Yersinia                    YGVADVPNYKLWV..SEPPLPDSVIDVGLRTEPNLELLTEMKPS......FMVWSAGYGP
Enterobacteriaceae          YGVADTINYRLWV..SEPPLPDSVIDVGLRTEPNLELLTEMKPS......FMVWSAGYGP
Salmonella                  YGVADTINYRLWV..SEPPLPDSVIDVGLRTEPNLELLTEMKPS......FMVWSAGYGP
Shigella                    LAVGDMQSYQTWV..KQPELPKTTVDLGVRLQPNLELIATLSHSADDLNLVFINSNFYAS
Shigella                    VGVADVKGYSAWV..TAGKLDSTPTDIGTRNEPSFDTIASLDPD......LIVATTDLAE
Haemophilus                 LAIADIRNYNTWV..VEPPLAPGVIDVGQRTEPNMELIQQLKPS......LLLITAGYGP
Micrococcaceae              LAIADIRNYNTWV..VEPPLAPGVIDVGQRTEPNMELIQQLKPS......LLLITAGYGP
Rahnella                    VGAADPEGYATWN..AAEALPGDVKDVGKRGEPSVDAIVALEPD......LVLMPLARDT
Rahnella                    VGVADVENYHKWV.DVREELSDDVVDVGLRTEPNLEAIAQLEPD......LIISANYRSE
Actinosynnema               VGVADPKGYATWV..TAAKLDPAVKDVGTRGEPSVDSIVALAPD......LVVVSDGRGA
Shouchella                  FGVADIHNYRLWV..GEPALPADVINVGQRTEPNLELLQQMAPS......LILLSQGYGP
Micromonospora              VGMADIKGFKAYV.KTPTPIAASVKDVGERGQVSLEVIAALKPD......LIINQSGDYS
Yersinia                    VAVGDVKSYQQWV..GEPALPNDTLDLGVRMQPNPELILTLKQ..GDHDLHFINSSFYAQ
Deinococcus                 VGVAQSKEFGTWN..TAVKLDKKVKDVGDRNEPSEDAISGLNPD......LVITTSDRPD
Actinobacillus              VGIADIAGMNKWV.NIPVEIGPDVTDVGTRQEPNLEMIASLKPD......LIIGLKSTSR
Stackebrandtia              VGIADIAGMNKWV.NIPVEIGPDVTDVGTRQEPNLEMIASLKPD......LIIGLKSTSR
Paenibacillus               VAMPDISGYREWV..MQPEVPDSVLDIGSRVEPNLQRLASLKPD......VIIIASPQLD
Paenibacillus               QGVADAQGYQQWV..VKPALNADVTDVGSRREPNLELLTELKPD......VILISEHMAA
Shewanella                  VAACELIRFRKDA..PEPPVPEGVVDLGLRGAPNFELLQLVRPD......LILTSPYYTR
Vibrio                      LAATDLDSYREWV..AQPPIPAQTQSVGSREEPNLERIAALKPD......VILASEMQQA
Cereibacter                 TGVADVKGYGEWYGAIDPKLDATVVDVGTRQEPSIETITSLEPD......LIIGVNFRHE
Edwardsiella                AGVADLDGYRNWV.NAGKSLSDSVEDVGTRQEPNLEAISRLDPD......LIIGVKFRHE
Paenibacillus               VAVADPKGYATYV..KAETLPADVADAGQRGEPDLDALYATNPD......LIVVEAFKAD
Halobacillus                VGNADNANYRLWI.TPEAKLADSVTDIGTRSEPNLEAIAALKPD......LIISNTDNNA
Actinoplanes                IGVADAAGYRQWV..VEPTLPQAVTDVGSRREPNLDLLTELKPD......VILISKHMAA
Paenibacillus               IAAPNLSGYRQWV..VNPYAPETIEDIGTRAEPNLEKIASLNPD......VILAASPQQD
Vibrio                      VGVADVKGYTAWN..TAAPLTGSPKDIGTRGEPSIDTVAALAPD......LVVATSDLSP
Vibrio                      VGAPELASYQDWV..VKPQVPQGVEDIGTRVEPNLEKIAALKPD......LILAAAPQQD
Streptomyces                VGQADVAGYNTWD..TVVPLDASVADVGMRGEPSLDAIAALDAD......LVVTTTDLPE
Vibrio                      LAMPEIEGYSEWV..VRPAISDTTEDIGSRAEPNLEKLTALQPD......VIVITPTLLD
Cellulomonas                VGVADPKGYATWV..TAAPLDASVKDVGTRGEPSVDSIVALQPD......LVVMEAERGS
Marinomonas                 VGNADNANYKVYV.TPEAALDSNVVDVGTRNEPNLEAIAALKPD......LIISNFDGNE
Micromonospora              EAVAQVDAYHAWV..GEPHLPGSVTDLGLRTQPNLELLSDLAPD......QILISPMFAN
Paenibacillus               VGVADPKGYATWV..TAAPLDASVKDVGTRGEPSVDSIVALQPD......LVVMEAERGS
Halomonas                   IAIPNPGEWADWV..VEPALPPGIANLGTDREPNLEVLAALKPD......LIVSTPYLDG
Micromonospora              AGMADIRGYHEWV.KIPVALDRSVPDVGTRQQPSLERIAAIKPD......LILAPSFRIT
Ancylobacter                VGVADVKGYSTWV..TAAKLDPSVKDVGTRGEASVDAIVGLDPD......LVVVTTDESA
Meiothermus                 VAVADVAGYTTWN..DAEKLPEGVTDVGTRGEPNLDTLYAADPD......LVIVEAYTPD
Kribbella                   VGQADVAGYNTWD..TSIALDEDTPDVGTRGEASLEAISALEPD......LIAVTTDTTD
Xylanimonas                 VGIADIEGMKQWV.NIPVEIGSDVTDVGTRQEPNLELIASLKPD......LIIGLKSNLE
Sanguibacter                VGVSLPDWYRSSI..VEPPLPPGVADIGLLYQPNFEVLLALRPD......LLVITPGHLP
Paenibacillus               LGIADIPNMKKWV.KLPVEIAPEVVDVGSRTSPNLESIMMLKPD......LIIGIKRNVE
Burkholderia                VAVGDVKSYQQWV..GEPALPNDTLDLGVRMQPNPELILTLKQ..SDHDLHFINSSFYAQ
Brevibacillus               VGVADVAGYSAWS..SAVPLVNEPADIGTRGEPSVETIASLAPD......LIVATTDLPA
Actinobacillus              VGVADVKGYSAWV..TAGKLDSTPTDIGTRNEPSFDTIASLDPD......LIVATTDLAE
Clavibacter                 VGVADIQGYKKYV.NVQPSLDESVVDVGTRQEPSLEAIAQLKPD......LILGVDLRHQ
Paenarthrobacter            EGVANISGYQQWV..AEPHLNANAIDVGSRREPNLELLSNIKPD......VILISKHLAA
Nostocaceae                 EGVANISGYQQWV..AEPHLNADAIDVGSRREPNLELLSNIKPD......VILISKHLAA
Vibrio                      VGVADIQGYKQYV.NVQPSLTESVVDVGTRQEPNLEAIAQLKPD......LILGVELRHK
Vibrio                      VGIPLPDWYASGI..VTPPLPPGVADVGLLYQPNFEVLLALAPD......LIIVTPAHAA
Nostocaceae                 LAVGDMQSYQTWV..KQPELPKTTVDLGVRLQPNLELIATLSHSADDLNLVFINSNFYAS
Burkholderia                VAVGDIKSYQQWV..GEPALPNDTLDLGVRMQPNPELILTLKQ..GDHDLHFINSSFYAQ
Haemophilus                 VGVADVKGYTAYN..TAAPLAKGIKDIGTRGEPSVATVASLKPD......LILATTDLSD
Actinobacillus              VGAPELSSYVQWV..VQPEVPSSVQDIGTRTEPNLEKIAALKPD......VILAAGPQQD
Streptomyces                VGAPELSSYVQWV..VQPEVPSSVQDIGTRTEPNLEKIAALKPD......FILAAGPQQD
Vibrio                      LAVADIHNYNLWV..EEPKLPASVIDVGQRTEPNLELLQQLQPS......LMLLSKGYGP
Vibrio                      YGVADVPNYKLWV..SEPPLPDSVIDVGLRTEPNLELLTEMKPS......FMVWSAGYGP
Vibrio                      FGVADVHNYNIWV..GQPALPAGVVDVGLRTEPNLELMTQMHPS......LILCSNGYGP
Serratia                    YGVADIPNYRLWV..NEPRLPDSVIDIGLRTEPNLELLTQMKPS......FLFWSAGYGP
Salmonella                  FAVADVPNYRLWV..DYPPLPDSVIDVGLRTEPNLELLTQLQPS......LMIWSAGYGP
Pantoea                     YGVADIPNYNLWV..NEPKIPDSVIDIGLRTEPNLELLTQMKPS......YLVWSAGYGP
Enterobacter                FAIADKHNYNLWV..KEPALPDSVIDVGLRTEPNLELLTQIRPS......MILYSEGYGP
Cronobacter                 FAIADKHNYNLWV..KEPALPGSVIDVGLRTEPNLELLTQIRPS......MILYSEGYGP
Enterobacter                LAIADIRNYNIWV..AEPPLGPGVIDVGQRTEPNMELIQQLNPS......LLLITTGYGP
Pectobacterium              YGVADIPNYRLWV..NEPALPDSVIDIGLRTEPNLELLTQMKPS......FLFWSAGYGP
Pectobacterium              YAVADVPNYRLWV..DYPPLPDAVIDVGLRTEPNLELLTELKPS......LMIWSAGYGP
Rahnella                    FGVADVHNYNIWV..GQPALPAGVVDVGLRTEPNLELMTQMHPS......LILCSNGYGP
Klebsiella                  LGAAELHNYRLWV..GKPELPPSVVDVGLRTEPNLELITQMQPS......LILFSQGYGP
Enterobacter                YGVADIPNYNLWV..NEPKLPASVIDIGLRTEPNLELLTQMKPS......FLFWSAGYGP
Cronobacter                 LAVADIHNYNLWV..EEPKMPASVIDVGQRTEPNLELLQQLQPS......LMLLSKGYGP
Pantoea                     YGVADTHNYRLWV..EEPELPTSVIDVGQRTEPNLELLQQMAPS......LILMSEGFGP
Erwinia                     LGVADMHNYRVWV..GEPQLPATTIDLGLRTEPNLELMTQLNPS......LILHSNGYGP
Klebsiella                  YGVADIPNYRRWV..SEPPLPASAIDVGLRTEPNLELLTEMQPS......FLVWSSGYGP
```

```
Enterobacter            MGVADIHNYRLWV..QAPALPTSVVDVGLRTEPNLELLARLNPS......LLLYSTGYGP
Serratia                MGAAELYNYGLWV..GEPALPASTVDVGLRTEPNIELMIQMKPS......LLLYSSGYGP
Yersinia                YGVADIPNYRLWV..NEPPLPASVIDVGLRTEPNLELLTEMKPS......FMVWSAGYGP
Pantoea                 LAIADKRNYNLWV..KEPELPESVIDIGLRTEPNLELLAQIRPS......MIIYSEGYGP
Enterobacter            LGAAELHNYRLWV..GKPELPPSVVDVGLRTEPNLELITQMQPS......LILFSQGYGP
Dickeya                 LGAAELHNYRLWV..GKPELPPSVVDVGLRTEPNLELITQMQPS......LILYSQGYGP
Erwinia                 YAVADVPNYRLWV..DYPPLPDAVIDVGLRTEPNLELLTELKPS......LMIWSAGYGP
Klebsiella              YGVADTHNYRLWV..EEPELPTSVIDVGQRTEPNLELLQQMAPS......LILMSEGFGP
Citrobacter             YGVADIPNYRLWV..NEPALPDSVIDVGLRTEPNLELLTQMKPS......FLVWSAGYGP
Pectobacterium          LGAAELYNYRLWV..GKPELPPSVVDVGLRSEPNLELITQMQPS......LILFSQGYGP
Erwinia                 YGVADIPNYRLWV..NEPALPDSVIDVGLRTEPNLELLTQMKPS......FIVWSAGYGP
Erwinia                 LAVADTSGYRSWV..GQPALPQSVVDLGLRTEPNLELLAQLKPS......LMVYSSGFGP
Cronobacter             YGVADTINYRLWV..SEPPLPDSVIDVGLRTEPNLELLTEMKPS......FMVWSAGYGP
Yersinia                YGVADTINYRLWV..SEPPLPDSVIDVGLRTEPNLELLTEMKPS......FMVWSAGYGP
Klebsiella              YGVADTINYRLWV..SEPPLPESVIDVGLRTEPNLELLTEMKPS......FMVWSAGYGP
Klebsiella              YGVADVPNYKLWV..SEPPLPDSVIDVGLRTEPNLELLTEMKPS......FMVWSAGYGP
Shimwellia              VAVP.KPIVPSTV..PNFLNNDQIVDLGTHREPNLEAMVATDPD......LIISGQRFSQ
Escherichia             VGAPELSSYVQWV..VQPEVPSSVQDIGTRTEPNLEKIAALKPD......VILAAGPQQD
Enterobacteriaceae      YGVADVPNYKLWV..SEPPLPDSVIDVGLRTEPNLELLTEMKPS......FMVWSAGYGP
Enterobacteriaceae      YGVADVPNYKLWV..SEPPLPDSVIDVGLRTEPNLELLTEMKPS......FMVWSAGYGP
Escherichia             YGVADVPNYKLWV..SEPPLPDSVIDVGLRTEPNLELLTEMKPS......FMVWSAGYGP
Escherichia             YGVADTINYRLWV..SEPPLPDSVIDVGLRTEPNLELLTEMKPS......FMVWSAGYGP
Escherichia             YGVADTINYRLWV..SEPPLPDSVIDVGLRTEPNLELLTEMKPS......FMVWSAGYGP
Salmonella              YGVADTINYRLWV..SEPPLPDSVIDVGLRTEPNLELLTEMKPS......FMVWSAGYGP
Salmonella              YGVADTINYRLWV..SEPPLPESVIDVGLRTEPNLELLTEMKPS......FMVWSAGYGP
Salmonella              YGVADIPNYRLWV..NEPALPDSVIDVGLRTEPNLELLTQMKPS......FIVWSAGYGP
Salmonella              YGVADTINYRLWV..SEPPLPDSVIDVGLRTEPNLELLTEMKPS......FMVWSAGYGP
Salmonella              KGLP.KSNIPSYL..SKYKD.DKYIDVGTLFEPNFEKLNEIKPD......VIFISARQSK
Salmonella              YGVADTINYRLWV..SEPPLPDSVIDVGLRTEPNLELLTEMKPS......FMVWSAGYGP
Enterobacteriaceae      KGLP.KSNIPSYL..SKYKD.DKYIDVGTLFEPNFEKLNEIKPD......VIFISARQSK
Enterobacteriaceae      YGVADTINYRLWV..SEPPLPDSVIDVGLRTEPNLELLTEMKPS......FMVWSAGYGP
Enterobacteriaceae      KGLP.KSNIPSYL..SKYKD.DKYIDVGTLFEPNFEKLNEIKPD......VIFISARQSK
Escherichia             YGVADVPNYKLWV..SEPPLPDSVIDVGLRTEPNLELLTEMKPS......FMVWSAGYGP
Klebsiella              TGVPTVAKWPPQL..SQYAD.QRYLKVGSMFEPNYEVIHAAAPQ......LIFVAGRSAP
Clostridium             YGVADVPNYKLWV..SEPPLPDSVIDVGLRTEPNLELLTEMKPS......FMVWSAGYGP
Clostridium             KGLP.KSNIPSYL..SKYKD.DKYIDVGTLFEPNFEKLNEIKPD......VIFISARQSK
Clostridium             YGVADIPNYRLWV..NEPALPDSVIDVGLRTEPNLELLTQMKPS......FLVWSAGYGP
Achromobacter           KGLP.KSNIPSYL..SKYKD.DKYIDVGTLFEPNFEKLNEIKPD......VIFISARQSK
Clostridium             YGVADIPNYRLWV..NEPALPASVIDVGLRTEPNLELLTQMKPS......FIVWSAGYGP
Clostridium             YGVADTINYRLWV..SEPPLPDSVIDVGLRTEPNLELLTEMKPS......FMVWSAGYGP
Shigella                YGVADIPNYRLWV..NEPALPDSVIDVGLRTEPNLELLTQMKPS......FIVWSAGYGP
Bacillus                VGSVIGGDLKDFLP.HAKEQLKDTKKLGVVTDPNMEALLQLKPS......EIYVDEKYAG
consensus>70            .gvad...y..wv....p.l...v.dvg.r.epnle....l.p................
```

```
                              α5       β5                   α6                    α7
Erwinia                                                                                 
                               110       120            130       140       150
Erwinia                   ..DPARLQRIAPGMGFAFNDG...SGKPLTGARHALMALANRIDRVPQAQAHLAQLDALM
Cereibacter               ..YEARLSQLAPVLNLPFYIR...GEPPLPKTFAALHNLARAIDDPGAALRAEEAAEARF
Rhizobium/Agrobacterium   ..YTGRLEAIAPVFSLPFYVK...GEPPFEKALAAVTALGEKLGRAEEARKVLGETEAAL
Azorhizobium              ..ISHRLERIAPVLSFPMYVP...DGQPLALATEALSRLAALTGRVSQGMAALAAVEQDF
Microlunatus              GPDGAKLAAIAPTFTYDGFDG.......DWRDDFKAVAAAFGKEQVAEDFLQQVNTRT
Rhodococcus               WYGTERLSSIAPLLVVGDDVEP..IAGHPVDWRGALMAQASQLDRVAIAEASIAEYDDKL
Beutenbergia              .TVIDQIATS.VPVLALRGSNA...EDPLGHMERTLDLLGQATGTQEQADELWSGFQTAL
Gottschalkia              ..VYEQLSKIAPTIVFDSFAP.........LEDRLSELGDILGKKQEADNWLKEYNKKL
Corynebacterium           ..AANLLERIAPTYVVSVYKQ...GSRPFEKASGMLTTLGEMLNREERAKAVLNDIDQTL
Marinobacter              ..HYKEMKKLNPGVPIVDFEPRDG.EAFDAELKRQVTEMGVIFNKEGEAKKLVKDFEDAV
Corynebacterium           ..HYKEMKKLNPGVPIVDFEPRDG.EAFDAELKRQVTEMGVIFNKEGEAKKLVKDFEDAV
Corynebacterium           ..IKPLLERVAPVTTIGIYTP...DGQPYQRAIEATRQLAALVGRESEGEALIAATEAHF
Mesorhizobium             ..HYEALSKIAP...TIYMGRDSHPDGLIASMKANTEILHQLYPGRDLEG.LMTDLESQV
Erysipelothrix            ..HQARLEKIAPVTAISIYAE...GKANWQALQDFTHTMASAIGKEDAAQQLIAASENSF
Marinomonas               ..MRAQLARIAPVTTYGSLSQ...EHDSYLVARDDYLALAQRFGREDHALRELAAMQVQI
Pseudomonas               ..LAPTLSRIAPVSTIALYDG...QTDLWQRLHEVTLTLARMVGKTAEGERLLVGLDRDL
Aeromonas                 ..VLRALSNIAPTEVVNIFPT...TQGVWNSASAFMRQLAERLHLSERADVYLQAAEQRL
Pseudomonas               ..ARARLERIAPVRSLALSGP...DGEAYANARRVARELAGLLGREAAGEALIARVDARL
Azotobacter               ..MYEKLNTIAPTLVYTIYND...KKAPLSSAKDITAQLGVLFNKEKQSAKVIAATEQRL
Aliivibrio                ..MYEKLSTIAPTLVYTVYSN...KKSPLIAAKGITIQLGRLLDKEQQALQLIDQTEKKL
Aliivibrio                ..LLGQFGRIAPTEVAHIFDG...CADPYLRIRELLLQMGTATGLEAQAQMRLREFDTEI
Alcaligenaceae            ..DNYILK..NKTKVIDFYQN...KKDVYSSINESVLQIGMILNKKQEALELIRNTKLFF
Campylobacter             ..IQPRLERIAPVFTRALYVA...DEPAFPKVMALLAELAQRLGDPAAGPRAQAAAQAEF
Paracoccus                ..LKPSLERIAPTSTVALYTP...GSDLWTRLEKATREIAAFVNREERADTLLASLEAHL
Chromohalobacter          ..IEPSLTAKAPVSTVNFYQ....PGDTWQNIENATRQIGELIEKSEQAEQLITQTNTQL
Mannheimia                ..MKHRLVDIAPVTTIDLYES...GITDWSVLTRFTRRFGEELGREAEAEALIQQYEERF
Vibrio                    ..LAPTLSRIAPVQSIALYDP...QTDLWQRLHEATLTIAALVNKTAEANVLLTDLNREL
Aeromonas                 ..LKPRLEPIAPVLELTIYAE...GGEALPRAIEATRTLATAIGREPQAADFLARSEQFF
Rhizobium                 ..SPEKLARIAPGRGFTFSD....GKRPLAMAQRSLLEMADLLGKTQQAKSHLAEFDALM
Klebsiella                ..SPEKLAPIAPTMSFAFNEQ...GSSPLAVGKNSLQTLGQRLGLETAAQQHLADFDHFM
Yersinia                  ..SPEKLARIAPGRGFDFSD....GKKPLAVARRSLVELAQTLNLEAAAEKHLAQYDRFI
Enterobacteriaceae        ..SSEMLARIAPGRGFNFSD....GKHPLAMARKSLTEMADLLNLQSAAETHLAQYEDFI
Salmonella                ..SAEMLARIAPGRGFNFSD....GKQPLAMARKSLTEMADLLNLQSAAETHLAQYEDFI
Shigella                  ..LNNLLRPYAQVHNVDFYQ....AGDAWQNVLNATRTIGQIIGKPQAVQQLLAQYEQDV
Shigella                  .PVIKQLEEL.APVVVVKSADG...SRQIAQAEDNLKLVAKATGTETKADEAIAAFDAAV
Haemophilus               ..TTSQLESIAPVLGVKANDG...SAKPLNLAISSLHKLADRLGLQDRAETHLQLYRDTL
Micrococcaceae            ..TTSQLESIAPVLGVKANDG...SAKPLNLAISSLHKLADRLGLQDRAETHLQLYRDTL
Rahnella                  .AVAEQLEKA.VPVVMTKSSDA...TKNFDRLREDFTLIAKAVGKEAEAKTALAEMDAKL
Rahnella                  .NIEEELKDIAPMLIFDPYPEESKGITHYEEMETTFREMAKVVDKEAEGEAILAELDEKY
Actinosynnema             .TLLDQLEKY.VPVLVTKSSDA...TDNVGRMRADLNMIAKAVGKTAEAEKLLADFDAAL
Shouchella                ..SPEKLAPIAPTMSFAFNEQ...GSSPLAVGKNSLQTLGQRLGLETAAQQHLADFDHFM
Micromonospora            .QRD.QLARIAPTLVFNPYPSAATPQTAYQEMRQTFALMGRITGRSAQATRVLAQLDAEQ
Yersinia                  ..ATATLEPFSSVTLVDFYT....EGDAWQNIVNASRKVAFIADKQAEFEALMTSYWQKI
Deinococcus               .STLKQLEKS.VPVLVVKGSDAK..SGNLKRMESDLDMIAEAVGRTDEADTLMEDFESKL
Actinobacillus            .TNYESLKGIAPTLVFDPYPPEG.AGDQYGEMISTFQTIADVLGKKDEAAKVLSSLDRTY
Stackebrandtia            .TNYESLKGIAPTLVFDPYPPEG.AGDQYGEMISTFQTIADVLGKKDEAAKVLSSLDQTY
Paenibacillus             ..LLPRLEKIAPVLFYHTYSE...HHDNSQAAIDNFKLIAQTLGKEAQAELRLVKMQQNI
Paenibacillus             ..AYHQLNKIAPVLVYSVYSK...KKQPLESATSVTLSLGKLFNKAQRAQQVIDETNKRL
Shewanella                ..YEARLSQLAPVLNLPFYIR...GEPPLPKTFAALHDLARAIDDPGAASRAEEAAEARF
Vibrio                    ..LLPRLMQIAPVLYYTNFSA...ADDHAAVAIAQFRQLAQAFGREAIAEQKLAAMQARF
Cereibacter               .AIYDQLSAIAPTLIFNPYPENG.GLDQYSEMEQTFSAIADALGKKAEGEKVLADLHSYY
Edwardsiella              .AIADQLEDIAPTVMFAPYSEEA.AKDQYQHMLDEFNTVAKITSKEDKAKEVVKNMEKTF
Paenibacillus             DELITKLEKRGVPVLATVGADA...SGQIANMKKVFSMIGTATGRTERADLVLQQFDKHL
Halobacillus              .AIYAQLKGIAPTIEYDFFKGKG...YDYDRMVEIFKQIAVATGKTEQADKVLNDLDQHY
Actinoplanes              ..AYAQLNKIAPVLVYTLYDD...QRAPLQAAQSATRSLGQLLDKQAQAEHVIEQTKQRL
Paenibacillus             ..LIPLLSQIAPVIYLPNFSE...NEAAAETAIRHFRTLGKLLDKQDLVEKKLTALDQSF
Vibrio                    .AVVKQLRKV.APVIEITSADA...ADQIGTMTEGLDLIAKATGKESESAALKKDFDTSV
Vibrio                    ..LITPLERIAPVLYFSNFQA...SDDSGAVAIDHFRQLAQLFDKQALAEQKLTTMTERF
Streptomyces              .EVIAQIEEQ.VPVIALRGSDA...SDPIGHMRRTVEVLGEATGTSDASEKALADFDAAV
Vibrio                    ..LRERLSDIAPVLVFDTYRK...DHNNMDAADKTFLQLGRLVGREEIAQQRLVEREQLF
Cellulomonas              .AVVTQLEKY.VPVLVTKGSDA...SDNIGRMRADLKMIATATGRTAQADKLLADFDAAL
Marinomonas               .AIYEQLKAIAPTIEYNLNDGNG...YDYDKMVEIFNSIAVALGKEDKAKQVLADLDQHY
Micromonospora            ..LKPRLSRIAPVQNFTLYTP...GRDTWQEMRELTHGVAELVGRPEAADRLIESTEARI
Paenibacillus             .AVVTQLEKY.VPVLVTKGSDA...SDNIGRMRADLKMIATATGRTAQADKLLADFDAAL
Halomonas                 ..IKLLLERFAPTRTFSVYAPP..VGSAYARSISATRALAADVGRRDAGEELIARAQATM
Micromonospora            .QTYGRLSQIAPTIAFNPFPEDGSSQ..FARVLQDFQTVAELVGKGDQGRRVRVQMEARF
Ancylobacter              .STIAQIEKA.ATVLVVRGADA...ANPIPQLKKNLELLGAAVGKSAQAAEANAAFDRAL
Meiothermus               DEIIATLEAYGVPVLATKGADA...ADPIANMFATFDLIAQATGRTDRAEAVAEEFEAHL
Kribbella                 .AVVDQLEDI.APVVVLRGSDG...TDPIGYMRSTVETLAAATGTDARGEELLASFDAKV
Xylanimonas               .AGFETYNSIAPTLVFDPYPAEG.QGDQYEEMINTFNTIADVLDKKNEAANVLSNLDQTY
Sanguibacter              ..ALRTLQRVAPTITLSQYMT...SAQPYRDLCGETVTLGARLGRTARAQQLVAEAARTT
Paenibacillus             .ANYDEMSKIAPTIAFDTNPTEG.QGSQYDRMIEIFKQIADITGKNAEAEAALKDLDKTY
Burkholderia              ..ATATLEPFSSVTLVDFYT....EGDAWQNIVNASRKVAFIADKQVEFEALMTSYWQKI
Brevibacillus             .DAITQLKAI.APVLQVNSADG...SKQIQQSEDNLELIAKATGTEDKATEVIGAYDQAV
Actinobacillus            .PVIKQLEEL.APVVVVKSADG...SRQIAQAEDNLKLVAKATGTETEADEAIAAFDAAV
Clavibacter               .TIYDTLSAIAPTLIFNPYPSAE.NSNQLDEMQQTFRKIAESVNRRDVGEKVLQQMQTQF
Paenarthrobacter          ..AYEPLSKIAPVLVYSVYSE...DKQPLESAKRITRSLGKLFDKEQQAEQVIAQTDQRL
Nostocaceae               ..AYEPLSKIAPVLVYSVYSE...DKQPLESAKRITRSLGKLFDKEQQAEQVIAQTDQRL
Vibrio                    .TIYDTLSAIAPTLIFNPYPSAE.NSNQLDEMQQTFRKIAERVNRQDVGEKVLQQMQTQF
Vibrio                    ..LMTPLRRIAPTLTLGAYMS...AARPLEVLCDETSAMARAIDAPLHAPELIAATQREF
Nostocaceae               ..LNNLLRPYAQVHNVDFYQ....AGDAWQNVLNATRTIGQIIGKPQAVQQLLAQYEQDV
Burkholderia              ..ATVTLEPFSSVTLVDFYT....EGDAWQNIVNASRKVAFIADKQVEFEALMTSYWQKM
Haemophilus               .SAIAQLSKA.APVAVVRSADA...SRQIDQMVDTVELIGKATGNEDKAESEVDSFRKAV
Actinobacillus            ..LLATLGRIAPVVYLPNFAE...QDNAAQVAVSHFKTLATLFGKEAVAQQKLEAMYARF
Streptomyces              ..LLATLGRIAPVVYLPNFAE...QDNAAQVAISHFKTLATLFGKEAVAQQKLEAMYARF
Vibrio                    ..TPQKLEPIAPSMSFSFNDG...SGKPLTVARQSLQALAQRLGLETRAVQHLAQFDRFI
Vibrio                    ..SPEKLARIAPGRGFDFSD....GKKPLAVARRSLVELAQTLNLEAAAEKHLAQYDRFI
Vibrio                    ..PKEKLQRIAPIMGFDLHGG...DGKPLSRARVSLRALGARLGREAQAEAHLQYVDAEI
Serratia                  ..SEETMARIAPGRGFSFSD....GKNPLAMAKNSLHEMALFLNREVEAKKHLDDFDALI
Salmonella                ..AEAQLKRIAPGMGVTFTD....GKAPLAMARQSLVALAARLGLESAAQAHLDEFERFL
Pantoea                   ..SPETLARIAPGRGFAFSD....GKKPLTMARHSLSEMAQLLDLEAAAKAHLDHFDSTI
Enterobacter              ..SVAKMSRISPLLGFGFSSE...QGKPLTSAQTSVMKLADALNMKAAGERHLTELAQFL
Cronobacter               ..SVAKMSRISPMLGFGFSSE...QGKPLTAAQSSVMKLADALNMKAAGERHLTELAQFL
Enterobacter              ..TTRQLETIAPVLDVKANDG...SAKPLNLAISSLHKLADRLGLQDRAEAHLQLYRNTL
Pectobacterium            ..SEETMARIAPGRGFSFSD....GKKPLTMAKNSINEMAHFLNREAEAKQHFAEFDALI
Pectobacterium            ..SEAQLKRIAPGMGVTFTD....GKAPLAMARQSLLTLAERLGLEKAAHAHLDEFEQFL
Rahnella                  ..PKEKLQRIAPIMGFDLHGG...DGKPLSRARVSLRALGARLGREAQAEAHLQYVDAEI
Klebsiella                ..DPARFQRIAPGMGFAFNDG...SGKPLTGARHALMALANRIDRMAQAQAHLAQLDALM
Enterobacter              ..SEATMARIAPGRGFSFSD....GKKPLTMAKNSVNEMARFLNREAAAKQHLDEFDALI
Cronobacter               ..TPQKLAPIAPTMSFGFNDG...SGKPLTVGRNSLQQLAQRLGLEARAEQHLMQFDRFI
Pantoea                   ..SPEKLAPIAPSMSFAFSQQ...GSTPLAVGKNSLRALGERLGLEAAAEQHLNDFNQFM
Erwinia                   ..SADTLSRIAPTMGFDLNSG...DGKPLSTARQSLHALGARIGREAQAVQHLQYVDGVL
Klebsiella                ..SPEKLARIAPGYGVTFSN....GVNPLATARASLTALAQRLGREEQAQRHLHDFDSFI
```

```
Enterobacter             ..AAEQLERIAPSLGFAFSDG...NGKPLQAARQSLATLAERLEMNAAAERHLAAFDQFM
Serratia                 ..SVDTLNKIAPSMGFAFSDD...RGKPLTVARESLMQLARRLDRVPVAQKHLAEYDDFT
Yersinia                 ..SPEKLARIAPGRGFNFSD....GKQPLAVARQSLLELGQLLNLAPAATRHLSEFDQFI
Pantoea                  ..SVAKMSRISPMLGFSFSSE...QGKPLTSAQASLMKLADALNMKAVGERHLAELAQFL
Enterobacter             ..DPARFQRIAPGMGFAFNDG...SGKPLTGARHALMALANRIDRMAQAQAHLAQLDALM
Dickeya                  ..DPARLQRIAPHMGFTFNDG...SDKPLTSARHALMALAERIDRVPQAQAHLAQLDALM
Erwinia                  ..SEAQLKRIAPGMGVTFTD....GKAPLAMARQSLLTLAERLGLEKAAHAHLDEFEQFL
Klebsiella               ..SPEKLAPIAPSMSFAFSQQ...GSTPLVVGKNSLRALGERLGLEAAAEQHLNDFNQFM
Citrobacter              ..SAEKLARIAPGRGFTFSD....GKRPLMMAQQSLGEMADLIGRQREAKRHLAEFDALM
Pectobacterium           ..DPARLQRIAPGMGFAFNDG...SGKPLTGARHALMALANRIDRVPQAQAHLAQLDALM
Erwinia                  ..SPEKLARIAPGRGFTFSD....GKRPLAMAQRSLLEMADLLGKTQQAKRHLAEFDALM
Erwinia                  ..APEKIARIAPGFGVTFSD....GRNPLNAARASITALGARLGLEDRARAHLADFDGFI
Cronobacter              ..SPEMLARIAPGRGFNFSD....GKQPLAMARKSLTEMADLLNLQSAAETHLAHYEDFI
Yersinia                 ..SPEMLARIAPGRGFNFSD....GKQPLAMARKSLTEMADLLNLQSAAETHLAHYEDFI
Klebsiella               ..SPEMLARIAPGRGFNFSD....GKQPLAMARKSLTEMADLLNLQSAAETHLAQYEDFI
Klebsiella               ..SPEKLARIAPGRGFDFSD....GKKPLAVARRSLVELAQTLNLEAAAEKHLAQYDRFI
Shimwellia               ..HYKEMKKLNPGVPIVDFEPRDG.EAFDAELKRQVTEMGVIFNKEGEAKKLVKDFEDAV
Escherichia              ..LLATLGRIAPVVYLPNFSE...QDNAAQVAISHFKTLATLFGKEAVAQQKLEAMYARF
Enterobacteriaceae       ..SPEKLARIAPGRGFDFSD....GKKPLAVARRSLVELAQTLNLEAAAEKHLAQYDRFI
Enterobacteriaceae       ..SPEKLARIAPGHGFDFSD....GKKPLAVARRSLVELAQTLNLEAAAEKHLAQYDRFI
Escherichia              ..SPEKLARIAPGRGFDFSD....GKKPLAVARRSLVELAQTLNLEAAAEKHLAHYDSVI
Escherichia              ..SPEMLARIAPGRGFNFSD....GKQPLAMARKSLTEMADLLNLQSAAETHLAHYEDFI
Escherichia              ..SPDMLARIAPGRGFNFSD....GKQPLAMARKSLTEMADLLNLQSAAETHLAQYEDFI
Salmonella               ..SPEMLARIAPGRGFNFSD....GKQPLAMARKSLTEMADLLNLQSAAETHLAHYEDFI
Salmonella               ..SPEMLARIAPGRGFNFSD....GKQPLAMARKSLTEMADLLNLQSAAETHLAQYEDFI
Salmonella               ..SPEKLARIAPGRGFTFSD....GKRPLAMAQRSLLEMADLLGKTQQAKRHLAEFDALM
Salmonella               ..SPEMLARIAPGRGFNFSD....GKQPLAMARKSLTEMADLLNLQSAAETHLAQYEDFI
Salmonella               ..AYEELNKIAP...TIHLDTENG..KYMESVKTNLDKLGKIFDKEDFVKDEIKKLDDSV
Salmonella               ..SSEMLARIAPGRGFNFSD....GKQPLAMARKSLTEMADLLNLQSAAETHLAQYEDFI
Enterobacteriaceae       ..AYEELNKIAP...TIHLNTENG..KYMESVKSNLEKLGKVFDKEDFVRDEIKKLDDSV
Enterobacteriaceae       ..SPEMLARIAPGRGFNFSD....GKQPLAMARKSLTEMADLLNLQSAAETHLTHYEDFI
Enterobacteriaceae       ..AYEELNKIAP...TIHLNTENG..KYMESVKSNLEKLGKVFDKEDFVRDEIKKLDDSV
Escherichia              ..SPEKLARIAPGRGFDFSD....GKKPLAVARRSLVELAQTLNLEAAAEKHLAQYDRFI
Klebsiella               ..KYDELAKLAP...TVDLTVNAQ..DLVGSVTRNTETLAAIYGKQDVAKTKLDALRASI
Clostridium              ..SPEKLARIAPGRGFDFSD....GKKPLAVARRSLVELAQTLNLEAAAEKHLAQYDRFI
Clostridium              ..AYEELNKIAP...TIHLNTENG..KYMESVKSNLEKLGKVFDKEDFVRDEIKKLDDSV
Clostridium              ..SAEKLARIAPGRGFTFSD....GKRPLMMAQQSLGEMADLIGRQREAKRHLAEFDALM
Achromobacter            ..AYQELNKIAP...TIHFNTENG..KYMESVKSNLEKLGKVFDKEDFVRDEIKKLDDSV
Clostridium              ..SPEKLARIAPGRGFTFSD....GKRPLAMAQRSLLEMADLLGKQQQAKRHLAEFDALM
Clostridium              ..SSEMLARIAPGRGFNFSD....GKHPLAMARKSLTEMADLLNLQSAAETHLAQYEDFI
Shigella                 ..SPEKLARIAPGRGFTFSD....GKRPLAMAQRSLLEMADLLSKTQQAKRHLAEFDALM
Bacillus                 .KDLAKYEKIAKTHSFNLDEG........TWRDHLKQVGKLVNREKEADTYIQDYEEQA
consensus>70             ......l..iap.........................la........a...l...d...
```

```
                                                   β6                β7             α8
Erwinia
                               160           170            180            190            200
Erwinia                       QQVKQRLATRA...HRPLLLMSIL..DARHAIVFTANGLFQ.EVMDHLGLENAWKG....
Cereibacter                   DAVAADLAP...MADRPICLVNIG..DARHLRAFGFDSLFG.SVATRLGLTNGWEG....
Rhizobium/Agrobacterium       HAMRARLAG...FSARPTYVINIG..DARHFRAFGADSMFG.DVLGRLGLTNAWVD....
Azorhizobium                  ASARSRLSG...TDRRPLLMVSLM..DSRHVRIYGANSLFG.AVLTRLDVANAYAG....
Microlunatus                  GEVKAKVAALG...EAPTVAYGYYYPGGNGEFIGSEPDNLIGQIFAEVGIKPPKAVG...
Rhodococcus                   ARLRPMLQEKLG..GKKIVLGVQT....PTGFLVHQRNLRI.SVAIDAGLDVLFRD....
Beutenbergia                  EDGAAALADAGLDGATFTMADGWNDAGVVSVRMFTTGSMLG.AVAGELGLVNGWE.....
Gottschalkia                  SHMWQQLIEDG.TIQPGETATVLTYYPGNRLFIMGTTGLSQ.VLYSPQGFKPGDKIQK..
Corynebacterium               QTQRRRLENAG.LTERPVALVNFL..DVRHVRVYAPNGLFQ.SALDALGLENAWPH....
Marinobacter                  ARA.KKAYNGSD...KVMAVNVSG..GAIGYIAPHVGRVYG.PVFDLLGLKPALQIEG..
Corynebacterium               ARA.KKAYNGSD...KVMAVNVSG..GAIGYIAPHVGRVYG.PVFDLLGLKPALQIEG..
Corynebacterium               SDVRQRLAP...LSARPIYVVSFL..DPRNVRVYGEKSLFQ.AVFDRIGIRNAWTG....
Mesorhizobium                 DAFKNKALKQTG...TLLFLMANG..NEIKAFGP..ESRYD.HVYRDFGFT..PVSKQ..
Erysipelothrix                QQLKASLPV...KPQADLLMIQFM..DARHVRVFGHNSMYQ.IALNQLGIKNAWSG....
Marinomonas                   DGLHQRLVEHFRGHLPKVLVIRFV..APGMVRVDGGDSMID.SALQQLHLQPAYPP....
Pseudomonas                   ASMQARLP....ADLPPLLVVQFI..DERHVRVFGRHSLFD.AVVTRLGLRNGWQE....
Aeromonas                     EVLRTRLALRPQPA..LYIAVLNQ..DGRHATVYGQHSMAQ.AVLDRLGLRNAWSG....
Pseudomonas                   AALRARLAG...RQRPPLYMAGFL..DARHVRLFGARSLYQ.GVLERVGLENAWNG....
Azotobacter                   LANGDNVHRLQ.SNSKPFLFLRFV..NDKTVRIHSEGSLTQ.DTIDAMGLVNAWHE....
Aliivibrio                    IENGDKIRLLQ.QQEKPLLFVRFI..NDKTVRIHSEGSLAQ.STINAMGLTNAWHE....
Aliivibrio                    ECLRAQLAAGADAARSVYMAVLHE..NGAQAFVYGQGSWVN.RVLGQLGLRNAWSA....
Alcaligenaceae                KSFKNKLH.....YAKPIAIVQFI..DRKYLRIYTKHSLFG.AVLEQLGLNNAYDK....
Campylobacter                 ATLAGRAAG...FADRACLLFELG..DSRHIRVFGTDSLFS.GALEAMGLRNAWTA....
Paracoccus                    AELRDGLSDA..AKARPLLVVQFM..DARHVRVFGDHSLYN.AVMQRLGLENAWQR....
Chromohalobacter              AKIGQTLTH...FRDRPVAIVQFI..DTRHLRFYDSHSLFG.TILNKLGLTNAWNH....
Mannheimia                    SLLKQRIN....KDQPPLLMIQFM..DTKHVRVFGQNSLYS.QAIEKIGLSNAWNE....
Vibrio                        EQMKQTLP....AELPPLLVVQFI..DERHVRVFGRHSLFE.AVMQRLGLRNAWQG....
Aeromonas                     DDCARRVQR...MSPSPLALVSFV..DQHHARIYGGAGLYQ.NVMTRIGLKNAWTG....
Rhizobium                     ESLRPRFAGRG...DRPHVLVFGENCLFQ.EVLDRFGIKNAWHG....
Klebsiella                    LAARARLSGDT...QTPLLMFSLL..DPRHALIIGNGSLFQ.DVLSTLNIENAWQG....
Yersinia                      ASQKPHFIRRG...GRPLLMTTLI..DPRHMLVLGPNCLFQ.EVLDEYGIVNAWQG....
Enterobacteriaceae            RSMKPRFVKRG...ARPLLLTTLI..DPRHMLVFGPNSLFQ.EILDEYGIPNAWQG....
Salmonella                    RSMKPRFVKRG...ARPLLLTTLI..DPRHMLVFGPNSLFQ.EILDEYGIPNAWQG....
Shigella                      QRLKTELSA...FTDRPLALLQFI..DTRHLRIYGENSLFG.AVAKQLGFRNAYLP....
Shigella                      TKGKAELEKAGVGGSRVAFADGWVADGKVSIRPFTKGSLLA.DINTELGLVNPWT.....
Haemophilus                   LQTREQLKPVA...QQPLLLITFI..DTRHVLVFGMGSMFH.EVMGELGLTNAWAG....
Micrococcaceae                LQTREQLKPVA...QQPLLLITFI..DTRHVLVFGMGSMFH.EVMGELGLTNAWAG....
Rahnella                      AEGKKAVEAKGATGTPFVMADGWMQGSTVSIRPFGKGSLVS.DTAEAVGLVNAWT.....
Rahnella                      EEGKEKIESMDLETRDFVLTMAYTDNQAPVFRLFNPNGMAP.AILEKIGLNNAYDS....
Actinosynnema                 ADGKKKIADAGAAGKPFAIADGWKEGSTVSIRMFGQGAFVS.QLGIQLGLTNAWN.....
Shouchella                    LAARARLSGDT...QTPLLMFSLL..DPRHALIIGNGSLFQ.DVLSTLNIENAWQG....
Micromonospora                AAARRTLTAAGRAGQTFVLSQGYT.YNTPTMRLFGTRSLAS.EILERTGLRNAYRP....
Yersinia                      DEIRPLVQP...YLERPIALVQFI..DTRHLRIYAANSPFG.AVLSQLGFHNAWSG....
Deinococcus                   AEGKKALGDA..ADTPFLMADGWDADGKISIRVHSTGSMYD.EVATGLGLKNAWT.....
Actinobacillus                ADAKAKLTAAGMTERPFALAMAYSNQNAVTFRISTDNSQAV.KILEHAGLKNAYKS....
Stackebrandtia                ADAKAKLTAAGMTERPFALAMAYSNQNAVTFRISTDNSQAV.KILEHAGLKNAYKS....
Paenibacillus                 AQMRTNLLAAYLGQLPKVTTFRFA..SVTSIYQFGDNSTAQ.YALSLLGIEPAIEQ....
Paenibacillus                 SDNGEKIRAAG.NSDKPLIFARFI..NDKTLRIHSQGSLAQ.ATISSMGLKNDWQE....
Shewanella                    DAVAADLAP...MADRPICLVNIG..DARHLRAFGFDSLFG.SVATRLDLANGWEG....
Vibrio                        QHLSRALRAAFQGQLPAVTVMRFA..STTSTYLYTTNSMPV.YVLRQLGISPALTP....
Cereibacter                   DESKAKLASAGKEGQEVVFTQAWTNEGVATFRLFTDNSMAM.AILAKIGLKNAHQD....
Edwardsiella                  RDQQARITEAGKEDSEYVMTQAFTSQNTPTLRLFTPNSIVS.KVMKNIGLQNAHQP....
Paenibacillus                 AEAKQKVANAAAR..DFLFFDGWIEGGNVVIRPYGEGALFT.ELGEQLGMTPAWTEKINA
Halobacillus                  IEAKATLEKADKADFHYALTQAYTAQNAASLRMFKDNSLVV.ETLAKIGMVNDWKS....
Actinoplanes                  AANGDKVRMAG.AADKPLLFVRFI..NDKTLRIHSQGSLAD.ATIRAMGLKNDWQE....
Paenibacillus                 DQMRLRIHQHYPDPL.EVLVMRFS..TPNTVFLSTENSTTD.YVVKHLGLNNPIHE....
Vibrio                        AEGKKALADAGLGGAEIASADGYVASNQVSVRAYTGGSLLG.AVNEKLGLKNAWT.....
Vibrio                        AQLKAQIGIAFSHQTPAVVAMRFA..NPTSVFLYGENSTTH.YVLKQLGLNEALPQ....
Streptomyces                  EDAKAELDEAGLAGARFTMADGWLSDGAVSVRMYTEGSYLG.GVAALLGLENAWT.....
Vibrio                        QNLTSKLRTKFGEQLPEVTTVRFA..STSSVWVYGDNAMPT.YVLERLGLKNGFSL....
Cellulomonas                  ADGKKKIADAGAAGKQFAIADGWKEGSTVSIRMFGQGALVS.QLGIQLGLKNAWT.....
Marinomonas                   AEAKQKLAAADKSDFHFILTQAFTSQNAASLRMFSDNSVVV.GTLAKIGLVNDWKP....
Micromonospora                DELRRQLP....DDVPPLLVVQFM..DARHVRVFGDNGLYQ.AVLERLGLDNAWPG....
Paenibacillus                 ADGKKKIADAGAAGKQFAIADGWKEGSTVSIRMFGQGALVS.QLGIQLGLKNAWT.....
Halomonas                     EETRRQLATAG.LAGRSLLVVNFL..DTRHVRVYGKGSLFG.DVMERTGLVNSWTK....
Micromonospora                AQARQLLQQAKRSGESFVLVQAFTSQNVGTMRLFTRNSIAS.EILERIGLKNAWS.....
Ancylobacter                  ADGRKKIADAGKAGENFTMADGWKQGSSISIRMFTTGSMLG.AIAGELGLKNSWT.....
Meiothermus                   ADATQRIADAAPEHTDFVYFDGWVDGGNVALRPFGQGSLVG.ELGEALGLNNAWT.....
Kribbella                     ADAKTTLEEAGVDGAAFTMADGWITNGTVSVRMYTAGSFLG.AIADELGLENAWA.....
Xylanimonas                   ADAKAKLEAAGMTEKPFALAMGYSNQNAAVFRISTDNSLAV.KILEHIGLTNAHKP....
Sanguibacter                  EAVRARLAARPALLRHPLIVAELV..DDRHLRVYGRGSLFD.EMLARLGAANAAHPRDGG
Paenibacillus                 AEAKEKLTKAGADKVPFVLAMGYSSQNAVEFRLSTDNSTAA.SILINIGLTNKYKP....
Burkholderia                  NEIRPLVQP...YLERPIALVQFI..DTRHLRIYAANSPFG.AVLSQLGFRNAWNG....
Brevibacillus                 TDAKAKLDAAGLAGSKFLFADAYVDAGAVAIRPFGTGSLIG.DVTTELGLENAWT.....
Actinobacillus                TKGKAELEKAGVGGSRVAFADGWVADGKVSIRPFTKGSLLA.DINTELGLVNPWT.....
Clavibacter                   QTVATRIKNTQKPD....FVLGQFSDNAPQIRLFTDNSMAA.QILTAIGLKNAWK.....
Paenarthrobacter              TANGAKITSAG.KAEKPLLFARFI..NDKTLRIHSEGSLAQ.DTINAMGLKNDWQE....
Nostocaceae                   TANGAKITSAG.KADKPLLFARFI..NDKTLRIHSEGSLAQ.DTINAMGLKNDWQE....
Vibrio                        QTAATRIKNTQKPD....FVLGQFSDNAPQIRLFTDNSMAT.QILTAIGLKNAWK.....
Vibrio                        ASAAARIADIGPHAVRAVIVTEAL..DERHLRVYGTGSLFD.DVLARIGVANAAHPRGAA
Nostocaceae                   QRLKTELSA...FTDLPLALLQFI..DTRHLRIYGENSLFG.AVAKQLGFRNAYLP....
Burkholderia                  GEIRPLVQP...YLERPIALVQFI..DTRHLRIYAANSPFG.AVLSQLGFHNAWGG....
Haemophilus                   ADGKKKLADAGLDGEKVAFADGWQEGNQVSVRPYVKGSLLS.DVNTELGLVDPWK.....
Actinobacillus                AELKASLQHAFGDTLPAVVTLRFA..NPTSVFLYTENSTPQ.YVLEQLGLSSALPQ....
Streptomyces                  AELKASLQHAFGDTLPAVVTLRFA..NPTSVFLYTENSTPQ.YVLEQLGLSSALPQ....
Vibrio                        QDARQRLHSYT...TRPLLMFSLI..DTRHALVIGQKSLFQ.EVMDQLGIINAWEG....
Vibrio                        ASQKPHFIRRG...GRPLLMTTLI..DPRHMLVLGPNCLFQ.EVLDEYGIVNAWQG....
Vibrio                        AAARQRLAPYA...GRAILLMSLL..DSRHAITFGKNSLFL.EVMERLGLKNAWQG....
Serratia                      NALKPRFAHRG...DRPLLMVTLL..DARHMLVFGKNCLFQ.DVLDSFGIRNAWEG....
Salmonella                    AQMKLRFAGRG...QRPLLMMSLL..DSRHALTLGTNSLFQ.PVLDAVNIPNAWQD....
Pantoea                       DALKPRFASRG...DRPLLMVTLL..DARHMLVFGKNCLFQ.EVLDRYGIPNAWEG....
Enterobacter                  QQEKITLQPYT...ERPLLLILI..DSRHVLIIAKNSLFQ.EVMDHVGIENAWQG....
Cronobacter                   QQEKITLQPYT...QRPLLLITLV..DSRHVLVIGKNSLFQ.EVMDHIGIENAWRG....
Enterobacter                  KQTREQLKPVA...HQPLLLITFI..DTRHVLVFGVGSMFH.EVMGELGLTNAWVG....
Pectobacterium                DSLKPRFAHRG...DRPLLMVTLL..DARHMLVFGKNCLFQ.EVLDSFGIRNAWEG....
Pectobacterium                TQMKARLAGRG...QRPLLMMSLL..DSRHALTLGTNSLFQ.PVLDAVKIPNAWQD....
Rahnella                      AAARQRLAPYA...GRAILLMSLL..DSRHAITFGKNSLFL.EVMERLGLKNAWQG....
Klebsiella                    QQVKARLAGRS...HRPLLLMSIL..DARHAIVFTANGLFQ.EVMDHLGLENAWKG....
Enterobacter                  DSLKPRFAHRG...ERPLLMVTLL..DARHMLVFGQNCLFQ.EVLDSFGIRNAWDG....
Cronobacter                   QDARQRLHSYT...RQPLLLFSLL..DTRHVLIIGQKSLFQ.EVMEQLGIHNAWEG....
Pantoea                       QAARQRFSASR...NTSLLMFSLL..DSRHALVIGQGSLFQ.DVLNELNIKNAWQG....
Erwinia                       AAARERLQPWA...GRPILLMSLL..DSRHAITFGKGSLFL.EVMDHLGLQSAWQG....
Klebsiella                    EQRRAGFARQ....RRPLLLVSLL..DARHMLVFGKNCLFQ.PVLDAYGLVNAWQG....
```

**Enterobacter**	AEARVRLQPYT...RRPLLLFSVL..DERHVLVVGKNSLFQ.DVLDQLGIENAWQG....
**Serratia**	AQMKNQLSYRP...KRPVLLMSVL..DPRHTLVFGKGSLFL.QVMGDLGLENAWQG....
**Yersinia**	ASRKPRFARRG...ERPLLLITQL..DARHMLVFGPNCLFQ.AVLDEYGIRNAWQG....
**Pantoea**	QQEKITLQPYT...QRPLLLITLI..DSRHALVIGKNSLFQ.EVMDHIGIENAWHG....
**Enterobacter**	QQVKARLAGRS...HRPLLLMSIL..DARHAIVFTANGLFQ.EVMDHLGLENAWKG....
**Dickeya**	QQVKTRLAGRS...PRPLLLMSIL..DARHAIVFTANGLFQ.EVMDHLGLENAWQG....
**Erwinia**	TQMKARLAGRG...QRPLLMMSLL..DSRHALTLGTNSLFQ.PVLDAVKIPNAWQD....
**Klebsiella**	QAARQRFSASR...NTSLLMFSLL..DSRHALVIGQGSLFQ.DVLNELNIKNAWQG....
**Citrobacter**	ESLRPRFARRG...DRPLLMITLL..DSRHVLVFAQNSLFQ.EVLDRFAIKNAWQG....
**Pectobacterium**	QQVKQRLATRA...HRPLLLMSIL..DARHAIVFTANGLFQ.EVMDHLGLENAWKG....
**Erwinia**	ESLRPRFAGRG...DRPLLMISLL..DPRHVLVFGENCLFQ.EVLDRFGIKNAWHG....
**Erwinia**	RQMAPRFAGR....QRPVLLMSLL..DPRHALVIGQHSLFQ.QVMDLLGIENAWQG....
**Cronobacter**	RSMKPRFVKRG...ARPLLLTTLI..DPRHMLVFGPNSLFQ.EILDEYGIPNAWQG....
**Yersinia**	RSMKPRFVKRG...ARPLLLTTLI..DPRHMLVFGPNSLFQ.EILDEYGIPNAWQG....
**Klebsiella**	RSMNPRFVKRG...ARPLLLTTLI..DPRHMLVFGPNSLFQ.EILDEYGIPNAWQG....
**Klebsiella**	ASQKPHFIRRG...GRPLLMTTLI..DPRHMLVLGPNCLFQ.EVLDEYGIVNAWQG....
**Shimwellia**	ARA.KKAYNGSD...KVMAVNVSG..GAIGYIAPHVGRVYG.PVFDLLGLKPALQIEG..
**Escherichia**	SELKASLQHAFGDTLPAVVTLRFA..NPTSVFLYTENSTPQ.YVLEQLGLSSALPQ....
**Enterobacteriaceae**	ASQKPRFIRRG...GRPLLMTTLI..DPRHMLVLGPNCLFQ.EVLDEYGIVNAWQG....
**Enterobacteriaceae**	ASQKPHFIRRG...GRPLLMTTLI..DPRHMLVLGPNCLFQ.EVLDEYGIVNAWQG....
**Escherichia**	ASQKPHFIRRG...GRPLLMTTLI..DPRHMLVLGPNCLFQ.EVLDEYGIVNAWQG....
**Escherichia**	RSMKPRFVKRG...ERPLLLTTLI..DPRHMLVFGPNSLFQ.EILDEYGIPNAWQG....
**Escherichia**	RSMKPRFVKRG...ARPLLLTTLI..DPRHMLVFGPNSLFQ.EILDEYGIPNAWQG....
**Salmonella**	RSMKPRFVKRG...ARPLLLTTLI..DPRHMLVFGPNSLFQ.EILDEYGIPNAWQG....
**Salmonella**	RSMNPRFVKRG...ARPLLLTTLI..DPRHMLVFGPNSLFQ.EILDEYGIPNAWQG....
**Salmonella**	ESLRPRFAGRG...DRPLLMISLL..DPRHVLVFGENCLFQ.EVLDRFGIKNAWHG....
**Salmonella**	RSMKPRFVKRG...ARPLLLTTLI..DPRHMLVFGPNSLFQ.EILDEYGIPNAWQG....
**Salmonella**	KYINKKATESGK...KALVVLAND..GALSAYGK..GSRFG.IIHEELGLP..LSDEH..
**Salmonella**	RSMKPRFVKRG...ARPLLLTTLI..DPRHMLVFGPNSLFQ.EILDEYGIPNAWQG....
**Enterobacteriaceae**	KDINKKASEGGK...KALVILAND..GALSAYGK..GSRFG.IIHEELGFP..LSDEH..
**Enterobacteriaceae**	RSMKPRFVKRG...ARPLLLTTLI..DPRHMLVFGPNSLFQ.EILDEYGIPNAWQG....
**Enterobacteriaceae**	KDINKKASEDGK...KALVILAND..GALSAYGK..GSRFG.IIHEELGFP..LSDEH..
**Escherichia**	ASQKPHFIRRG...GRPLLMTTLI..DPRHMLVLGPNCLFQ.EVLDEYSIVNAWQG....
**Klebsiella**	ADLNGKAASAG....TALIVLTTG..GKMSAYGP..GSRFG.VIHDAFGIK..PATTG..
**Clostridium**	ASQKPHFIRRG...GRPLLMTTLI..DPRHMLVLGPNCLFQ.EVLDEYGIVNAWQG....
**Clostridium**	KDINKKASEGGK...KALVILAND..GALSAYGK..GSRFG.IIHEELGFP..LSDEH..
**Clostridium**	ESLRPRFARRG...DRPLLMITLL..DSRHVLVFAQNCLFQ.EVLDRFAIKNAWQG....
**Achromobacter**	KDINKKASEGGK...KALVILAND..GALSAYGK..GSRFG.IIHEELGFP..LSDEH..
**Clostridium**	ENLRPRFARRG...DRPLLMISLL..DARHVLVFGENCLFQ.EVLDRFGIQNAWRG....
**Clostridium**	RSMKPRFVKRG...ARPLLLTTLI..DPRHMLVFGPNSLFQ.EILDEYGIPNAWQG....
**Shigella**	ESLRPRFAGRG...DRPLLMISLL..DPRHVLVFGENCLFQ.EVLDRFGIKNAWHG....
**Bacillus**	KRVKSLIDKELG.NNEKVMAIRVT...AKELRVFSTKRPMGPILFQDLGLQPANGVEK..
consensus>70	..........................d..........sl....v....g..naw......

```
                                              TT   β8    η1           β9          α9       α10
Erwinia                                     210       220          230       240       250

Erwinia                        ........EKTFWGSAVIGIERLAKLG.....DVEAICFEHGNEK...LMQQVSASALW
Cereibacter                    ........QTRFSFLAPVQIEELARLPE.....ARVVIVGE.IPVQ...ARRGLARSKLW
Rhizobium/Agrobacterium        ........RSQFTFAAPVPLENLAASSD.....ARIVIVSD.IPVE...ARETLRNSAIW
Azorhizobium                   ........QTNFWGFTTLGIESLPPLAD.....ARLVFFDP.VPPG...TLAALAANPLW
Microlunatus                   .........KEWTEISAEKIPELSDVD.....LLMIAVETDSDPDK.ELAALRKDPIW
Rhodococcus                    ........DDNAENSFELSFEQLDTLAP......ADVIIAQARDQE...ALDAIKTQPTW
Beutenbergia                   ....GEG..DPDYGLAATDVEGLLALP..DDTHFVYLANDTDGGDP...FVDGLGTNPVW
Gottschalkia                   .......FLDEKKGFAEISLELLPEYAG....DRIFVLNPETNLHDAKESTAKMINSQLW
Corynebacterium                .......TGN.YWGFSVVGLEAIAPYQD.....SRIVVISPTLPG...LSDTLANSPFW
Marinobacter                   ........ASSNHKGDDISVEAIADSNPDWILVLDRDGAIMADDAKYTPAKDVIAKNQAL
Corynebacterium                ........ASSNHKGDDISVEAIADSNPDWILVLDRDGAIMADDAKYTPAKDVIAKNQAL
Corynebacterium                ........ETNYWGFATVGIDGLATSDD.....ARLAYLEP.LPEG...AGGTLTESQVW
Mesorhizobium                  ........FDVSTHGSTLSFEQIQDLNPDYILVMDR.SRVTGGEG....NAEVLMDNVFV
Erysipelothrix                 ........DTN.AWGFALVGIDSLLDIK......GQFVVVDPLPAG...AKEHLEQDQLW
Marinomonas                    ........PHD...AAVPIEALAGIE.......DGVVVYVEPFA...GQEQLFASPEW
Pseudomonas                    .......QTN.DWGFSVVSIEQFMTLPT.....ARLVVVDPIPVG...VSERLQEPGLW
Aeromonas                      ........PSNAMGFSLTGIERLSEQPDA....HLLYIEIPTTSAR...LQSLRQPNALW
Pseudomonas                    ........RTNRWGFAQVGIERLAERPE....ATLLYLES.LPPH...AARLRAASPLW
Azotobacter                    ........PTN.MWGFTTAGVEKLAQYQQ.....SNVLIFGPLKET...ERKQLTHSALW
Aliivibrio                     .......QTN.LWGFTTAGIEKLAQYQQ.....ANVLIFGPLKEE...EKVKLTQSALW
Aliivibrio                     ........RTTFYGNSLVGIAALAAEPEA....VILYLDQGARTRR...AEALLRDSTLW
Alcaligenaceae                 .......DVNNAWGVDTISFLDLLKFSK.....ETKFIVIKPTPFT...LDEELKKNPFY
Campylobacter                  ........RTSFAFAAPVPLEKLAEYPE.....ARLVIVGR.MPPQ...AERGLGSAALW
Paracoccus                     .......ETN.YWGFSMVGLEALAGLDD.....ARLVVVDPMPVG...VEERLETSALW
Chromohalobacter               .......SGG.VWGSENLSITALATLPK....NTRLVVVKPHPAN...VANALKYNSLW
Mannheimia                     .......ETN.SWGFSLVGIDKLAGIR......AQIVIVEPLPHG...GKKRLSQDPFW
Vibrio                         .......ETN.AWGFSVASIEQFMALPA.....ARLVVVNPIPVG...VSERLQEPGLW
Aeromonas                      ........SGNFWGFETIGIEQLASLDQN....LRLIVFEPLIPPN...ILSGLEESPLW
Rhizobium                      ........EAAFWGSVSVGIDRLAAFN......EADVICFDHGNER...DMAQLLATPLW
Klebsiella                     ........ETNFWGSAVVGIERLATIK......TARAVCFGHGNNE...MLQQVARTPLW
Yersinia                       ........ETNFWGSTAVSIDRLAMYK......EADVICFDHGNNT...DMNALMATPLW
Enterobacteriaceae             ........ETNFWGSTAVSIDRLAAYK......DVDVLCFDHDNSK...DMDALMATPLW
Salmonella                     ........ETNFWGSTAVSIDRLAAYK......DVDVLCFDHDNSK...DMDALMATPLW
Shigella                       .......KVN.YWGFQNIEITELAKLPP.....NTRFVIVKPYPAN...IASALTHNTLW
Shigella                       ....VEG..DPAYGLGSTDVEGLTAVN...ADHFVYITNSADGD.....FTEQLTDNAVW
Haemophilus                    ........EGSFWGSVSVGVERLTRIK......NARVLCFEHDNAD...MMEAVAKTPLW
Micrococcaceae                 ........EGSFWGSVSVGVERLTRIK......NARVLCFEHDNAD...MMEAVAKTPLW
Rahnella                       ....GEV..DAEWGLGATDVEGMTAIT...DPKTIMVYSASE.EDV...FTTGLKDNAVW
Rahnella                       ........GSFELYGYATTGVEELLSVE.....DANFLHIVQDDDGL...FEEGLANNAVW
Actinosynnema                  ....GKV..DEVWGLGQTDVEGLTALK...SPDLHFFYNASDGTDV...FADGLADNAIW
Shouchella                     ........ETNFWGSAVVGIERLATIK......TARAVCFGHGNNE...MLQQVARTPLW
Micromonospora                 ........DKASDYGFDTLSLEGLTTLK.....TQNFFAIAPEDDNV...FTASG.NRAVW
Yersinia                       .......SQN.AWGFETIDVTQLAKLAP.....NSRLVVVKPYPAN...IGSALRYNTLW
Deinococcus                    ....KKVAYDKVWGLDTVDVEALSYLE...DKKLKFMYDDSGKFGV...FTKKLKGNKIW
Actinobacillus                 .......EKFEIYGFTTTDVEALPALQ.....DANFLHIIQGSDNV...IENQLKDNPVW
Stackebrandtia                 .......EKFEIYGFTTTDVEALPALQ.....DANFLHIIQGSDNV...IENQLKDNPVW
Paenibacillus                  ........APTQWGVKQKRLKELHNID.......DGVALYFEPFA...QESQLKGSVMW
Paenibacillus                  .......QTN.LWGFTTTGIERLAEHQQ.....TNVMIFGPLTEE...EHKKLTQSPLW
Shewanella                     ........QTRFSFLAPVQIEELARLPE.....ARVVIVGE.IPVQ...ARRGLARSKLW
Vibrio                         ........PPARWGIVRQPIAGLQRVG.......QGYVLYFLPFH...DAARLDAMRLW
Cereibacter                    .......STFQQYGYSETDIEGLTKTP.....DASVLYTTSATDTV...FSELLPKNEVY
Edwardsiella                   .......DKTEVYGYTSTTVETLQNYQ.....DAHFFYIVQEEDNI...FTNQLEGNPAW
Paenibacillus                  A..YGSGGVDPSYGLAQTDIEGLTAVG.....NATLFYSDDATPDS...YVKELTKSPIW
Halobacillus                   .......DKTEKYGFSTVGIEALPAVQ.....DSHFIYITQKTDDV...FGAAMENNSVW
Actinoplanes                   .......ATN.LWGFTTAGIAKVAEHQH......SRVMIFGPLEPT...AHQQLTQSPLW
Paenibacillus                  ........SPKAWGVKQDRINRLQNLE.......DSYILYVQPFP...QEDKLKDSPLW
Vibrio                         ....VKG..DESYGLATTDVEGLTGLG...DVQFTYYVANDADG.DA...FAGPLAKNAVW
Vibrio                         ........PAKEWGIVQHRLTDLQYVE.......QGYVLYFLPFA...QQAQLEKSMLW
Streptomyces                   ....GEG..DPDYGLATTDVEGLTNLG..EDVHFLYVANDTEGGDA...FTEGLSGNAIW
Vibrio                         ........PVTQWGVTQIRIKKLNAIK.......EGYLLYFEPFH...QQQKLERSLLW
Cellulomonas                   ....GKT..DEMWGLGQTDVEGMTVLK...GQDVHFFYNASDGTDV...FADGLAGNAIW
Marinomonas                    .......SKVESYGFSTVGIEALSAVQ.....DSNFIYIVQPDDNV...FGSSMKNNSVW
Micromonospora                 .......KTN.AWGFSLTGIEALANLD.......ARLVVVEPYPAG...VHETLEKSGLW
Paenibacillus                  ....GKT..DEMWGLGQTDVEGMTVLK...GQDVHFFYNASDGTDV...FADGLAGNAIW
Halomonas                      ........PSNYWGFSTVGIEELADSPA.....SSLVYLEP.ISTD...TLDRLAASPLW
Micromonospora                 .......DRPQLYGYSTLGLEGLASIR.....ADNFFYIVQDDDNV...FLNPS.VEPLW
Ancylobacter                   ....GAG..DKDYGLAQTDIEGLTKLP...DGNFLYIASTTDGGDV...FGGDLAKNAIW
Meiothermus                    ......GEVDPAYGLGQSDIEGMSAVG.....DATFLYTGTQDGDD...WTASLAKNAVW
Kribbella                      ....GEG..DPVYGLAQTDIEGLTALSDTQDLTFLYAASDSED.DP...FADGLAGNAIW
Xylanimonas                    .......GQFEVYGFTTADVEALPALQ.....DANFLHIIQDSDNI...IENQLKDNPVW
Sanguibacter                   ......AAWPT.QAGYALVPLQRLAEVPA.....ASLLLVGP.VRPD...ARRGLDDNAIW
Paenibacillus                  .......KKFEQTGMTLADVEALPALQ.....DANFLHIIQNDDNV...IENQLKNNPVW
Burkholderia                   .......SQN.AWGFETIDVTQLAKLAP.....NSRLVVVKPYPAN...IGSALRYNTLW
Brevibacillus                  ....GEV..DPAYGLGSTDVEGLTTVG...DVQFLYNSNSTQGDDP...FASTLAGNAVW
Actinobacillus                 ....VEG..DPAYGLGSTDVEGLTAVN...ADHFVYITNNADGD.....FTEQLTDNAVW
Clavibacter                    .......GEFDRFGFNTVWVEALPKVE.....TANFIYISAP.NSP...YKQQLETNPVW
Paenarthrobacter               .......PTN.LWGFTTTGTEKLAEHQK......ANVMIFGPLSQE...ERQQLTQSPLW
Nostocaceae                    .......PTN.LWGFTTTGTEKLAEHQK......ANVMIFGPLSQE...ERQQLTQSPLW
Vibrio                         .......GEFDRFGFNTVWIEALPTVE.....TANFIYISAP.NSP...YKQQLETNPVW
Vibrio                         SRAVSAPWRTSVTGSATVALERLFDVPH.....ADILLIGP.LRAD...VRAALDQSPLW
Nostocaceae                    .......KVN.YWGFQNIEITELAKLPP.....NTRFVIVKPYPAN...IASALTHNTLW
Burkholderia                   .......SQN.AWGFETIDVTQLAKLAP.....NSRLVVVKPYPAN...IGSALRYNTLW
Haemophilus                    ....LKG..DAAYGLAATDVEGLTKIG...DARFAYIANDSDGGDP...FADGLKDNAVW
Actinobacillus                 ........PPKEWGIVQKRLSELQHVE.......QGYVLYFLPFA...EEKKVQKSVLW
Streptomyces                   ........PPKEWGIVQKRLSELQHVE.......QGYVLYFLPFA...EEKKVQKSVLW
Vibrio                         ........ETNFWGTAVVGIERLAVVK.....SARAIYLDHGNQE...MLKKVSSTPLW
Vibrio                         ........ETNFWGSTAVSIDRLAMYK......EADVICFDHGNNT...DMNALMATPLW
Vibrio                         ........ETNFWGSSVIGLERLAAFH......DVEAICFDHDNAA...VMQQLSRSPLW
Serratia                       ........EMTFWGSTAVGIDRLAAFR......DVDVLCFDHGNER...EMQTLMATPLW
Salmonella                     ........KTNFWGSAIVGIERLAQFR......DADVICFEHGDDQ...AMRQLAATPLW
Pantoea                        ........EMTFWGSTAIGIDRLAQFR......DVDVLCFDHGNER...EMQTLMATPLW
Enterobacter                   ........ETSFWGSTIVGIERLVEIG......DANVLCFEHGDSA...ISAQLDNNPLW
Cronobacter                    ........ETSFWGSTIVGIESLAEIG......DANVLCFEHGDSA...IIAQLDKNPLW
Enterobacter                   ........EGSFWGSVSVGVERLASIK......NARVLCFEHDNTD...MMDAVAKTPLW
Pectobacterium                 ........EMTFWGSTAVGIDRLAAFR......DVDVLCFDHGNER...EMQALMATPLW
Pectobacterium                 ........KTNFWGSAIVGVERLAQFR......DADVICFEHGDDQ...AMRQLAATPLW
Rahnella                       ........ETNFWGSSVIGLERLAAFH......DVEAICFDHDNAA...VMQQLSRSPLW
Klebsiella                     ........EKTFWGSAVIGIERLATMR......DVEVICFDHGNDN...LMQQVSASALW
Enterobacter                   ........EMTFWGSTTVGIDRLAAFR......DVDVLCFDHGNER...EMQKLMSTPLW
Cronobacter                    ........ETNFWGTAVVGIERLITVK......NARAIYLDHGNQA...MMEKVSSTPLW
Pantoea                        ........ETNIWGSAVVGIERLATVK......PGRAICFVHGNSK...MLQQVTRTPLW
Erwinia                        ........ETNFWGSAVIGLERLAAVG......DVPVICFDHDNEL...QMQEVMRTALW
Klebsiella                     ........KTHFWGSDVVGIDRLGEFK......DADVICFDHGNEP...EMQKLMATPLW
```

```
Enterobacter          ........STNFWGSTVVGMEQLASVG.....HANAICFEHGNEG...LMSRVSANPVW
Serratia              ........ETNFWGSAVIGIERLASIAE....DVDVVCFDHGEDL...MMAQVTSTPLW
Yersinia              ........ETNFWGSTVVGIDRLAAYK.....DADVLCFDHGDNK...EMDSLVRTPLW
Pantoea               ........ETSFWGSTIVGIESLANIG.....DANVLCFEHGDSA...ISAQLDSNPLW
Enterobacter          ........EKTFWGSAVVGIERLATMR.....DVEVICFEHGNDN...LMQQVTASALW
Dickeya               ........EKTFWGSAVIGIERLAILH.....NVEAICFEHGNES...LMQQVTASPLW
Erwinia               ........KTNFWGSAIVGVERLAQFR.....DADVICFEHGDDQ...AMRQLAATPLW
Klebsiella            ........ETNIWGSAVVGIERLATVK.....PGRAICFVHGNSE...MLQQVTRTPLW
Citrobacter           ........ETTFWGSVTVGIDRLAAYR.....DADVICFDHGNAR...EMAQLMATPLW
Pectobacterium        ........EKTFWGSAVIGIERLAKLG.....DVEAICFEHGNEK...LMQQVSASALW
Erwinia               ........EAAFWGSVSVGIDRLAAFN.....EADVICFDHGNER...DMAQLLATPLW
Erwinia               ........EVNFWGSSVVGIERLAEIR.....NADVICFDHDNDT...ITRQVMATPLW
Cronobacter           ........ETNFWGSTAVSIDRLAAYK.....DVDVLCFDHDNSK...DMDTLMATPLW
Yersinia              ........ETNFWGSTAVSIDRLAAYK.....DVDVLCFDHDNSK...DMDALMATPLW
Klebsiella            ........ETNFWGSTAVSIDRLAAYK.....DVDVLCFDHDNSK...DMDALMATPLW
Klebsiella            ........ETNFWGSTAVSIDRLAMYK.....EADVICFAHGNNT...DMNALMATPLW
Shimwellia            ........ASSNHKGDDISVEAIADSNPDWILVLDRDGAIMADDAKYTPAKDVIAKNQAL
Escherichia           ........PPKEWGIVQKRLSELQHVE.......QGYVLYFLPFA...EEKKVQKSVLW
Enterobacteriaceae    ........ETNFWGSTAVSIDRLAMYK.....EADVICFDHGNST...DMNALMATPLW
Enterobacteriaceae    ........ETNFWGSTAVSIDRLAMYK.....EADVICFDHGNST...DMNALMATPLW
Escherichia           ........ETNFWGSTAVSIDRLAMYK.....EADVICFDHGNNT...DMNALMATPLW
Escherichia           ........ETNFWGSTAVSIDRLAAYK.....DVDVLCFDHDNSK...DMDALMATPLW
Escherichia           ........ETNFWGSTAVSIDRLAAYK.....DVDVLCFDHDNSK...DMDALMATPLW
Salmonella            ........ETNFWGSTAVSIDRLAAYK.....DVDVLCFDHDNSK...DMDALMATPLW
Salmonella            ........ETNFWGSTAVSIDRLAAYK.....DVDVLCFDHDNSK...DMDALMATPLW
Salmonella            ........EAAFWGSVSVGIDRLAAFN.....EADVICFDHGNER...DMAQLLATPLW
Salmonella            ........ETNFWGSTAVSIDRLAAYK.....DVDVLCFDHDNSK...DMDALMATPLW
Salmonella            ........IDTAVHGQKISFEYVVEKNPDYIFVVDRGAVVQGGHK....SVNKILENDLI
Salmonella            ........ETNFWGSTAVSIDRLAAYK.....DVDVLCFDHDNSK...DMDALMATPLW
Enterobacteriaceae    ........IDTAVHGQKISFEYVVEKNPDYIFVVDRGAVVQGGHK....SVNKILENDLI
Enterobacteriaceae    ........ETNFWGSTAVSIDRLAAYK.....DVDVLCFDHDNSK...DMNALMATPLW
Enterobacteriaceae    ........IDTAVHGQKISFEYVVEKNPDYIFVVDRGAVVQGGHK....SVNKILENDLI
Escherichia           ........ETNFWGSTAVSIDRLAMYK.....EADVICFDHGNST...DMNALMATPLW
Klebsiella            ........LSVSNHGQAISFEFIAQTDPDWLFVIDRDAAIGREGV....SAQRMLDNELV
Clostridium           ........ETNFWGSTAVSIDRLAMYK.....EADVICFDHGNNT...DMNALMATPLW
Clostridium           ........IDTAVHGQKISFEYVVEKNPDYIFVVDRGAVVQGGHK....SVNKILENDLI
Clostridium           ........ETTFWGSVTVGIDRLAAYR.....DADVICFDHGNAR...EMAQLMATPLW
Achromobacter         ........IDTAVHGQKISFEYVVEKNPDYIFVVDRGAVVQGGHK....SVNKILENDLI
Clostridium           ........EAAFWGSVTVGIDRLAAFN.....EADVICFDHGNDR...EMAQLLATPLW
Clostridium           ........ETNFWGSTAVSIDRLAAYK.....DVDVLCFDHDNSK...DMDALMATPLW
Shigella              ........EAAFWGSVSVGINRLAAFN.....EADVICFDHGNER...DMAQLLATPLW
Bacillus              ........IDGNRPFEVISQEVLPDFD.....ADAIFVVVNRDDKAKAAFKQLQETLIW
consensus>70          .............g.....ie.l.............................l....lw
```

```
                             α11       β10                α12
Erwinia                      ᘐᘐ ᘐᘐᘐᘐ    ━━━▶      TT      ᘐᘐᘐᘐᘐᘐᘐᘐᘐᘐᘐᘐᘐᘐᘐ
                                 260        270            280        290

Erwinia                      QSMPFVRERRFRQVPR.VWFYG...ATLSAMRFIHCLDRVAGEE..............
Cereibacter                  QALPAVRGGRVYQLPE.VNAFG...GLPSALRFARMLGAALTRPAELRL.........
Rhizobium/Agrobacterium      RALPAVRENRVVTLAN.VGPYG...GITAGMRFARLLTEALLARGEAL..........
Azorhizobium                 TRLPYVQAGRVSRLPP.VLMFG...ALPSATRFARLLADALTGEHSHG..........
Microlunatus                 SQLPAVKAGHVVAVNNELSYAS....PHAHLEFLDVLEPNLALLAGE...........
Rhodococcus                  QRLPAVQAGNVLTSDA.RYNQG...FALTAAIFLDVLEEAAGRITAGQ..........
Beutenbergia                 TGLPFVESGQVYRLPDGIWQFG...GTASAEAFIDATVTALTS...............
Gottschalkia                 NDLPAVKNGHVYFIDIQKSNSD....ASTREWLLEEVPRMLKK...............
Corynebacterium              TYLPPVQRNQVYQIDP.VWPFG...GVFPVKRLATLLAEGLLAGGSDNVR........
Marinobacter                 AGVAAVKNQKVLVAPQDTYRNE...SIITYTKIFNSIADAFEQGK.............
Corynebacterium              AGVAAVKNQKVLVAPQDTYRNE...SIITYTKIFNSIADAFEQGK.............
Corynebacterium              NAMPFVRNRSIMRLAP.VLMFG...TLPSASRFARLLAQALTTEPAGG..........
Mesorhizobium                KATRAYQNGHIIYVDPEVWYLT.EGGSTAVLSMIESLKPLLP................
Erysipelothrix               QYLIKKSGHPVLRIEP.VWSFG...AIPSTVRFATLITHAIQEQEVN...........
Marinomonas                  RAMPFVRADRLAAMPD.TWAHG...GVFSIQYLAEAIAQALLSLPAP...........
Pseudomonas                  QHLPQVRQAPVLHLPA.VWSFG...GVLAARRFAGLLSDVLLQDAERMEASR......
Aeromonas                    NNLPAVRRNQTQALGK.FYPYG...GVASFLSLAERIADYLDRSEVRHG.........
Pseudomonas                  QRLPSVRAGRAHGLSP.VWSFG...GLPAAERFAGQLEAYLTALPSPDSDSPHP....
Azotobacter                  QAMAFTRTDSVYELPA.IWTFG...GLISAQRFSDHITQQLTQ...............
Aliivibrio                   QAMAFTRTGSVYELPA.IWTFG...GLISAQRFSDHITQQLTQ...............
Aliivibrio                   RSLPAVASGRAHAIAS.FYALG...GLASAQRCARLVVGALT..ERGHG.........
Alcaligenaceae               QKINIFQDY..NELDP.IWSAG...SLMSMRRFAKYLVEVIK................
Campylobacter                NSLPPVKAGRVHRLPE.LRPFA...GIPSALRFAHELVAALEGRA.............
Paracoccus                   QHLPSVRRDDVLTLPP.VWSFG...GAPSARRFADNLTQALHETPRTAAHREAPDA..
Chromohalobacter             RNLALAEDP..LLLPA.IWSFG...ALPSAVNFAQNLQSALLNQRSETW.........
Mannheimia                   QYVVQQSGEKVMQVAP.VWSFG...SMPSALRFAELVTASKVEELEQ...........
Vibrio                       QHLPLVQQAPVLHLPA.VWSFG...GVLAARRFATLLSEALQKDARGDRQ........
Aeromonas                    TSLPFVKAGRISVLPG.SLMFG...MVQEAERFARILLDHLEKAA.............
Rhizobium                    QAMPFVRAGRFQRVPA.VWFYG...ATLSAMHFARVLADAQGSPA.............
Klebsiella                   QSLSFVRENQLRLLPP.VWFYG...ATLSAMRFVRLLEQAWGKAP.............
Yersinia                     QAMPFVRAGRFHRVPA.VWFYG...ATLSTMHFVRILNNVLGGKA.............
Enterobacteriaceae           QAMPFVRTGRFQRVPA.VWFYG...ATLSAMHFVRVLDNAIGGKA.............
Salmonella                   QAMPFVRAGRFQRVPA.VWFYG...ATLSAMHFVRVLDNAIGGKA.............
Shigella                     QHLPMTREA..LILPA.VWSFG...AIPSARRFAHVFADGLLHG.GDAW.........
Shigella                     KSLPFVAAGNVHRLSDGIWMFG...GPASMTQYVDAIVASLTK...............
Haemophilus                  KSMPFVRANQFSIVPA.VWYYG...ATFSAMNFCKILTSTLGKPV.............
Micrococcaceae               KSMPFVRANQFSIVPA.VWYYG...ATFSAMNFCKILTSTLGKPV.............
Rahnella                     QRLPFTASGKITKLEKGTWTFG...GPKSVAVIADQFVKAATS...............
Rahnella                     NDLNFVKEGRVYALGGDTWPYG...GPLSAMTLIDRTVEALDQ...............
Actinosynnema                KSLPFVKQGNLHRMPDGIWTFG...GPLSAKQYIDQFVNTYAV...............
Shouchella                   QSLSFVRENQLRLLPP.VWFYG...ATLSAMRFVRLLEQAWGKAP.............
Micromonospora               NNLDFVKQGRGYSLDPATWVFG...GPYSAQVLIRQVVDVMTGK..............
Yersinia                     QHLAMAKDP..LILPA.VWTFG...GIPSAQRFAEMFANGLLHG.GEQW.........
Deinococcus                  DSLTFVKDGETHKLPDGIWMFG...GPKSGEQMIDAIVEVYTS...............
Actinobacillus               KGLNFVKENRVYALGGDMWPYG...GPLSAQVMAKKTAELLTP...............
Stackebrandtia               KGLNFVKENRVYALGGDMWPYG...GPLSAQVMAKKTAELLTP...............
Paenibacillus                NAMPFVRHQKMNSVKP.AWNYG...GAVSIEYMAQALTESLLEIAPDKPINPARESSQ
Paenibacillus                QAMAFTRTDSVYELPT.IWTFG...GLIAAQRFSDHITELLIQK..............
Shewanella                   QALPAVRGGRVYQLPE.VNAFG...GLPSALRFARLLGAALTRPAELRL.........
Vibrio                       QAMPFVRGQRVSAVRP.VWSYG...GAMSLQYAAEAMAESLMALAAQK..........
Cereibacter                  KNLNFAKENRVYDMKG.IWPFG...GPLSAKLLVERTLEALG................
Edwardsiella                 EDLKFVQEDRTYKMPGDTWTFG...GPLSAEVLAKQVTDAMLNENQSE..........
Paenibacillus                TALPAVKEKRAYAFPAGVWGAG...GPLSNEQAIDAYVKILTQA..............
Halobacillus                 KELNFVKEKRTYPLDGTTWTFG...GPISSKVLVDQVVGVLTK...............
Actinoplanes                 QAMAFTRSDSVYELPA.IWTFG...GLIAAQRFSDHITQQLIGQ..............
Paenibacillus                QAMPFVKKHRVNSVRA.VWAYG...GAMSLQYMAEAITDSLIELAPEQ..........
Vibrio                       KSLPFVKAGDVHRLPDGVWMFG...GPRSMEAYIDSLVDALTK...............
Vibrio                       QAMPFVRQARVNSVES.VWNYG...GAMSLQYIAEALTRSLLEVAPKS..........
Streptomyces                 QQLPFVTAGDVHRLPDGIWMFG...GPAAGEAWIDATVDALTK...............
Vibrio                       KAMPFVRHGKVNAIRS.TWTYG...GAMSLGYLAQALTDSLLDIAP............
Cellulomonas                 RSLPFVQQNKLHKMPNGIWTFG...GTLSAKQYIDELVKVYTA...............
Marinomonas                  NGLKFVKEKRTYPLASTTWTFG...GPVSSKGLVDGVVGAITQ...............
Micromonospora               QGLIRDSRGEPLVLPP.VWSFG...ALPSAQRFAEQLTAALESPHAR...........
Paenibacillus                RSLPFVQQNKLHKMPNGIWTFG...GTLSAKQYIDELVKVYTA...............
Halomonas                    NSLPFVKAGRIHRLPP.VLMFG...MLPSAMRFARQIARVLVEASSNG..........
Micromonospora               KNLEPVKNNRAYRLSGQTWTFG...GPLSAETLVNAVITAMLRR..............
Ancylobacter                 KNLPFVKSGNVHRLPDGIWMFG...GPKSMQQFIDATVQAVTS...............
Meiothermus                  ANLPSVVDGRTHAFPEHIWTFG...GPRSAEKIVDAYVDVFAR...............
Kribbella                    EQLPFVTAGDVHRIPDGIWMFG...GPASAEAYVDALVAALTA...............
Xylanimonas                  KGLNFVKEDRVYALGGDMWPYG...GPLSAQTMAQKTADLLTQ...............
Sanguibacter                 QALPAVRERRVATLPV.IAPYG...GLVSMQRFAGAIEAALTAIDAGGGGVA......
Paenibacillus                NGLTFVKENRVYALGGDLWPYG...GTMSAKILANKAVDLLAK...............
Burkholderia                 QRLAMAKDP..LILPA.VWTFG...GIPSAQRFAEMFANGLLHG.GEQW.........
Brevibacillus                QSLPFVTAGDVHRMPDGIWAFG...GPASMTAYAKAVSDLLAG...............
Actinobacillus               KSLPFVAAGNVHRLSDGIWMFG...GPASMTQYVDAIVASLTK...............
Clavibacter                  QKLQFVQQKRLYAIAPDTWLFG...GPLSAQILVDKIIKTLT................
Paenarthrobacter             QAMEFSRTDSVYELPA.IWTFG...GLLAAQRLSDHITGRLTQPQ.............
Nostocaceae                  QAMEFSRTDSVYELPA.IWTFG...GLLAAQRLSDHITGRLTQPQ.............
Vibrio                       QRLQFVQQKRLYAIAPDTWLFG...GPLSAQVLLQKVIDTLT................
Vibrio                       RALPAVREKRVAVLPV.IAAFG...GLVSMRRFAAAVSSALATLEHGGGGLA......
Nostocaceae                  QHLPMTREA..LILPA.VWSFG...AIPSARRFAHVFADGLLHG.GDAW.........
Burkholderia                 QHLAMAKDP..LILPA.VWTFG...GIPSAQRFAEMFANGLLHG.GEQW.........
Haemophilus                  KSLPFVKNDEVHRLPDGIWMFG...GTASMREYIDALVGALTA...............
Actinobacillus               RAMPFVQAGRVNSVRS.VWSYG...GAMSLRYSAEAITESLLAVAPQS..........
Streptomyces                 RAMPFVQAGRVNSVRS.VWSYG...GAMSLRYSAEAITESLLAVAPQS..........
Vibrio                       QALPFVRQNQLRQVPA.VWFYG...ATLSAMRFCHLLEQAQESYS.............
Vibrio                       QAMPFVRAGRFHRVPA.VWFYG...ATLSTMHFVRILNNVLGGKA.............
Vibrio                       QAMPFVQSGHFQRVPA.VWYYG...ATLSALHFIRVLEHALEKV..............
Serratia                     QAMPFVREKRFLRAPA.VWFYG...ATLSAMHFARVLDNALGGQA.............
Salmonella                   QAMPFVREDRFQRVPA.VWFYG...ATLSAMRFGRVLDNALGGRA.............
Pantoea                      QAMPFVREQRFQRAPA.VWFYG...ATLSAMHFARVLDAALGGKA.............
Enterobacter                 QAMPFVRQQRLQRVPA.VWLYG...GTLSAMRFCRILNQALEARNHA...........
Cronobacter                  QAMPFVRQRRVQRVPA.VWLYG...GTLSAMRFCRVLNQAMEARNHA...........
Enterobacter                 KSMPFVRANQFSIVPA.VWYYG...ATFSAMNFCEMLTRTLRKPV.............
Pectobacterium               QAMPFVREQRFTRAPA.VWFYG...ATLSAMHFARVLDSALGGKA.............
Pectobacterium               QAMPFVREGRFQRVPA.VWFYG...ATLSAMRFGRVLENALGGRA.............
Rahnella                     QAMPFVQSGHFQRVPA.VWYYG...ATLSALHFIRVLEHALEKV..............
Klebsiella                   QSMHFVRQRRFRQVPR.VWFYG...ATLSAMQFIHCLDRVLGDK..............
Enterobacter                 QAMPFVREQRFQRVPA.VWFYG...ATLSAMHFARVLDGALGGKA.............
Cronobacter                  QALPFVRQNQLRQVPA.VWFYG...ATLSAMRFCHLLEQTQESYS.............
Pantoea                      QSMSFVRQNQLQILPA.VWFYG...ATLSAMRFVRLLESAWGKSS.............
Erwinia                      QALPFIRAGHFQRVPA.VWYYG...ATYSALKFVRVLEHALESH..............
Klebsiella                   QAMPFVRQQRFLRLPP.VWFYG...ATLSVMHFVRLLERALGAKA.............
```

```
Enterobacter            QSLPFVRDRRWQRSET.VWLYG...ATLSAMRFCRLLDSAMERIT.............
Serratia                QSMPFVRKNRFHRVPQ.VWFYG...GTLSAMRFSRLLSSALVTA..............
Yersinia                QAMPFVRAGRFQRVPA.VWFYG...ATLSAMRFVRILDQALGGKA.............
Pantoea                 QAMPFVRQQRLQRVPA.VWLYG...GTLSAMRFCRVLNQALEAKKHA...........
Enterobacter            QSMPFVREQRFRQVPR.VWFYG...ATLSAMQFIHCLDRVLGDK..............
Dickeya                 QSMPFVRERRFRQVPR.VWFYG...ATLSAMQFLHCLDRELGEK..............
Erwinia                 QAMPFVREGRFQRVPA.VWFYG...ATLSAMRFGRVLENALGGRA.............
Klebsiella              QSMSFVRQNQLQILPA.VWFYG...ATLSAMRFVRLLESAWGKSS.............
Citrobacter             RAMPFVRAGRFQRVPA.VWFYG...ATLSAMHFARVLADAQGGPA.............
Pectobacterium          QSMPFVRERRFRQVPR.VWFYG...ATLSAMRFIHCLDRVAGEE..............
Erwinia                 QAMPFVRAGRFQRVPA.VWFYG...ATLSAMHFARVLADAQGSPA.............
Erwinia                 QALPFVRQRRFWRAPP.VWFYG...ATLSAMHFIRLIDKIAGGRA.............
Cronobacter             QAMPFVRAGRFQRVPA.VWFYG...ATLSAMHFVRILDNAIGGKA.............
Yersinia                QAMPFVRTGRFQRVPA.VWFYG...ATLSAMHFVRVLDNAIGGKA.............
Klebsiella              QAMPFVRAGRFQRVPA.VWFYG...ATLSAMHFVRVLDNAIGGKA.............
Klebsiella              QAMPFVRAGRFHRVPA.VWFYG...ATLSTMHFVRILNNVLGGKA.............
Shimwellia              AGVAAVKNQKVLVAPQDTYRNE...SIITYTKIFNSIADAFEQGK.............
Escherichia             RAMPFVQAGRVNSVRP.VWSYG...GAMSLRYSAEAITESLLAVAPQS..........
Enterobacteriaceae      QAMSFVRAGRFHRVPA.VWFYG...ATLSTMHFVRILNNVLGGKA.............
Enterobacteriaceae      QAMPFVRAGRFHRVPA.VWFYG...ATLSTMHFVRILDNVLGGKA.............
Escherichia             QAMPFVRAGRFHRVPA.VWFYG...ATLSTMHFVRILNNVLGGKA.............
Escherichia             QAMPFVRAGRFQRVPA.VWFYG...ATLSAMHFVRVLDNAIGGKA.............
Escherichia             QAMPFVRTGRFQRVPA.VWFYG...ATLSAMHFVRVLDNAIGGKA.............
Salmonella              QAMPFVRAGRFQRVPA.VWFYG...ATLSAMHFVRVLDNAIGGKA.............
Salmonella              QAMPFVRAGRFQRVPA.VWFYG...ATLSAMHFVRVLDNAIGGKA.............
Salmonella              QAMPFVRAGRFQRVPA.VWFYG...ATLSAMHFARVLADAQGSPA.............
Salmonella              QAMPFVRAGRFQRVPA.VWFYG...ATLSAMHFVRVLDNAIGGKA.............
Salmonella              KTTKAYKNNKIISLNPELWYIS.SGGIVSTTEMLKEIKDSIK................
Salmonella              QAMPFVRAGRFQRVPA.VWFYG...ATLSAMHFVRVLDNAIGGKA.............
Enterobacteriaceae      KTTKAYKNNKIISLNPELWYIS.SGGIVSTTEMLKEIKDSIK................
Enterobacteriaceae      QAMPFVRAGRFQRVPA.VWFYG...ATLSAMHFVRILDNAIGGKA.............
Enterobacteriaceae      KTTKAYKNNKIISLNPELWYIS.SGGIVSTTEMLKEIKDSIK................
Escherichia             QAMPFVRAGRFHRVPA.VWFYG...ATLSTMHFVRILNNVLGGKA.............
Klebsiella              RPTKAWKNQRVVYLNGYNWYLLGSAGLTAMQQNVDEIAGALAAGK.............
Clostridium             QAMPFVRAGRFHRVPA.VWFYG...ATLSTMHFVRILNNVLGGKA.............
Clostridium             KTTKAYKNNKIISLNPELWYIS.SGGIVSTTEMLKEIKDSIK................
Clostridium             RAMPFVRAGRFQRVPA.VWFYG...ATLSAMHFARVLADAQGGPA.............
Achromobacter           KTTKAYKNNKIISLNPELWYIS.SGGIVSTTEMLKEIKDSIK................
Clostridium             QAMPFVRAGRFQRVPA.VWFYG...ATLSAMHFARVLADAQGRAAV............
Clostridium             QAMPFVRTGRFQRVPA.VWFYG...ATLSAMHFVRVLDNAIGGKA.............
Shigella                QAMPFVRAGRFQRVPA.VWFYG...ATLSAMHFARVLADAQGRPA.............
Bacillus                KDLKAVKGKHVYIINDQPWLDY...SALGNKMAMDEAEKMFTK...............
consensus>70            ..m.fv...........vw.yg......s.............................
```
